# The active form of quinol-dependent nitric oxide reductase from *Neisseria meningitidis* is a dimer

**DOI:** 10.1107/S2052252520003656

**Published:** 2020-03-21

**Authors:** M. Arif M. Jamali, Chai C. Gopalasingam, Rachel M. Johnson, Takehiko Tosha, Kazumasa Muramoto, Stephen P. Muench, Svetlana V. Antonyuk, Yoshitsugu Shiro, Samar S. Hasnain

**Affiliations:** a Graduate School of Life Science, University of Hyogo, 3-2-1 Kouto, Kamigori, Ako, Hyogo 678-1297, Japan; b Molecular Biophysics Group, Institute of Integrative Biology, Faculty of Health and Life Sciences, University of Liverpool, Liverpool L69 7ZB, United Kingdom; c School of Biomedical Sciences, Faculty of Biological Sciences, University of Leeds, Leeds LS2 9JT, United Kingdom; d Astbury Centre for Structural and Molecular Biology, University of Leeds, Leeds LS2 9JT, United Kingdom; e RIKEN SPring-8 Center, 1-1-1 Kouto, Sayo, Hyogo 679-5148, Japan

**Keywords:** quinol-dependent nitric oxide reductase, *Neisseria meningitidis*, proton transfer, cryo-electron microscopy, oligomerization

## Abstract

*Neisseria meningitidis* is carried by nearly a billion humans, causing developmental impairment and over 100 000 deaths a year. A quinol-dependent nitric oxide reductase (qNOR) plays a critical role in the survival of the bacterium in the human host. X-ray crystallographic analyses of qNOR, including that from *N. meningitidis* (*Nm*qNOR) reported here at 3.15 Å resolution, show monomeric assemblies, despite the more active dimeric sample being used for crystallization. Cryo-electron microscopic analysis of the same chromatographic fraction of *Nm*qNOR, however, revealed a dimeric assembly at 3.06 Å resolution. It is shown that zinc (which is used in crystallization) binding near the dimer-stabilizing TMII region contributes to the disruption of the dimer. A similar destabilization is observed in the monomeric (∼85 kDa) cryo-EM structure of a mutant (Glu494Ala) qNOR from the opportunistic pathogen *Alcaligenes* (*Achromobacter*) *xylosoxidans*, which primarily migrates as a monomer. The monomer–dimer transition of qNORs seen in the cryo-EM and crystallographic structures has wider implications for structural studies of multimeric membrane proteins. X-ray crystallographic and cryo-EM structural analyses have been performed on the same chromatographic fraction of *Nm*qNOR to high resolution. This represents one of the first examples in which the two approaches have been used to reveal a monomeric assembly *in crystallo* and a dimeric assembly in vitrified cryo-EM grids. A number of factors have been identified that may trigger the destabilization of helices that are necessary to preserve the integrity of the dimer. These include zinc binding near the entry of the putative proton-transfer channel and the preservation of the conformational integrity of the active site. The mutation near the active site results in disruption of the active site, causing an additional destabilization of helices (TMIX and TMX) that flank the proton-transfer channel helices, creating an inert monomeric enzyme.

## Abstract 2

X-ray crystallographic and cryo-EM structural analyses have been performed on the same chromatographic fraction of quinol-dependent nitric oxide reductase from *Neisseria meningitidis* to high resolution. This represents one of the first examples in which the two approaches have been used to reveal a monomeric assembly in the crystal and a dimeric assembly in the frozen cryo-EM solution. A number of factors have been identified that may trigger the destabilization of the helices that are necessary to preserve the integrity of the dimer.

## 1. Introduction   

*Neisseria meningitidis* is carried by about 10% of the population of the world in a nonvirulent, asymptomatic form, which can develop into a pathogenic infection that affects over a million humans (Pizza & Rappuoli, 2015 ▸). A quinol-dependent nitric oxide reductase (qNOR) plays a critical role in the survival of the bacterium in the human host by combatting the host’s immune response. qNORs belong to the broader family of nitric oxide reductases (NORs). NORs are largely found as the essential component of anaerobic nitrate respiration, also known as denitrification, in the bacterial cytoplasmic membrane and catalyse the reduction of nitric oxide (NO) to nitrous oxide (N_2_O). There are three types of respiratory bacterial NORs: cytochrome *c*-dependent NORs (cNORs), dicopper NORs (Cu_*A*_NORs) and qNORs (Hendriks *et al.*, 2000 ▸; Hino *et al.*, 2010 ▸; Matsumoto *et al.*, 2012 ▸; Shiro *et al.*, 2012 ▸; Al-Attar & De Vries, 2015 ▸). Whilst cNORs are pre­dominantly found in denitrifying organisms as a respiratory enzyme, qNORs are also found in several pathogenic bacteria, including the Gram-negative, human-pathogenic bacterium *N. meningitidis*, as an enzyme that is responsible for the detoxification of NO produced by the host (Hendriks *et al.*, 2000 ▸; Anjum *et al.*, 2002 ▸; Rock & Moir, 2005 ▸). High-resolution structure determination of *N. meningitidis* qNOR (*Nm*qNOR) is of importance to provide a framework for developing a knowledge-based strategy for the design of new antibacterial agents, as well as to help to understand the chemistry of NO reduction, 2NO + 2 H^+^ + 2e^−^ → N_2_O + H_2_O, at the heme and nonheme iron (Fe_*B*_) binuclear centre.

NORs have also attracted attention with regard to the evolutionary aspects of respiratory enzymes. NORs are classified into the respiratory heme-copper oxidase (HCuO) superfamily, owing to the active site containing dinuclear metals in the form of a high-spin heme (heme *b*_3_) and either an iron or a copper ion (Sousa *et al.*, 2012 ▸). Pivotal to the HCuO mechanism is proton pumping across the membrane, coupled to the reduction of O_2_ to H_2_O, generating an electrochemical gradient which subsequently drives ATP synthase for ATP generation, which is termed an electrogenic reaction (Tsukihara *et al.*, 1996 ▸). Conflicting evidence has appeared for the electrogenicity of NORs; it has been suggested that cNORs are non-electrogenic (Hendriks *et al.*, 2000 ▸; Hino *et al.*, 2010 ▸, 2012 ▸), while increasing evidence has appeared for the electro­genicity of qNORs (Matsumoto *et al.*, 2012 ▸; Gonska *et al.*, 2018 ▸), similar to the Cu_*A*_NORs (Al-Attar & De Vries, 2015 ▸) there is proton pumping across the membrane during NO reduction.

All of the crystallographic structures have shown a monomeric assembly, despite the fact that all qNORs have been crystallized from the chromatographic peak that is assigned as a dimer. Very recently, we determined the structure of *Ax*qNOR by single-particle cryo-electron microscopy (cryo-EM) at 3.9 Å resolution (Gopalasingam *et al.*, 2019 ▸), showing it to be a dimer; this was the first dimeric structure of an NOR.

In order to elucidate the structural and functional properties of monomeric and dimeric forms of a qNOR, we focused on *Nm*qNOR, crystals of which were grown from the dimer fraction, providing a crystallographic structure at 3.15 Å resolution in which it was found to be a monomer. Contrary to this, cryo-EM analysis of the same fraction gave a dimeric structure at a resolution of 3.06 Å. To shed light on the monomer–dimer structural transition, we produced a number of point mutations of *Nm*qNOR and *Alcaligenes* (*Achromobacter*) *xylosoxidans* qNOR (*Ax*qNOR). The Glu498Ala mutant of *Nm*qNOR and the equivalent Glu494Ala mutant of *Ax*qNOR showed very low NO-reduction activity. We chose the Glu494Ala mutant of *Ax*qNOR, which primarily migrated as a monomer on a chromatographic column, to define a cryo-EM structure of a monomeric qNOR. This provided a 4.5 Å resolution structure of a monomeric qNOR for the first time, showing significant helical rearrangement compared with the dimeric cryo-EM structures of *Ax*qNOR and *Nm*qNOR, clearly indicating that the dimer–monomer transition is a key determinant of active NOR enzymes.

## 2. Methods   

### 2.1. Purification of recombinant *Nm*qNOR, *Nm*qNOR-BRIL and *Ax*qNOR Glu494Ala-BRIL   

Wild-type and site-directed mutants of *Nm*qNOR were overexpressed in *Escherichia coli* C43 or BL21 (DE3) strains (Lucigen) and were cultured in 2×YT and LB media with the addition of isopropyl β-d-1-thiogalactopyranoside (IPTG) to induce overexpression. The protein was purified using the detergents *n*-dodecyl-β-d-maltoside (DDM; Dojindo) and *n*-decyl-β-d-thiomaltoside (DTM; Anatrace), as previously reported (Gonska *et al.*, 2018 ▸). Apocytochrome *b*_562_ (BRIL; Chu *et al.*, 2002 ▸) was fused to the nontruncated C-terminus of *Nm*qNOR in order to increase the molecular weight to make cryo-EM analysis tractable. For cryo-EM analysis, *Nm*qNOR-BRIL was overexpressed and purified in a similar manner as for the non-BRIL construct, except that glycerol was excluded during the gel-filtration step. *Ax*qNOR Glu494Ala-BRIL was expressed and purified in a similar manner to the previously reported preparation of *Ax*qNOR Val495Ala-BRIL (Gopalasingam *et al.*, 2019 ▸).

### 2.2. UV–visible spectra measurement   

UV–visible spectra of *Nm*qNOR were measured using a Jasco V-630 spectrophotometer. All measurements were made using a quartz cuvette and the wave scans of samples were performed in the range 250–700 nm with a 1 nm step. The preparation of reduced qNOR was performed in an airtight quartz cuvette with a rubber seal. The sample was reduced by the addition of 5 m*M* sodium dithionite (Sigma–Aldrich).

### 2.3. Measurement of NO consumption by *Nm*qNOR and *Nm*qNOR-BRIL   

NO-reduction activity was determined using an NO electrode (World Precision Instruments) at 20°C under anaerobic conditions. Assay solutions (bubbled with N_2_ prior to use) contained 5 ml 50 m*M* HEPES pH 8.0, 150 m*M* NaCl and 0.05%(*w*/*v*) DDM or DTM (the latter was used for *Nm*qNOR-BRIL activity measurements). To maintain anaerobic conditions, 10 m*M* glucose, 50 µg ml^−1^ glucose oxidase and 50 µg ml^−1^ catalase were added to the reaction mixture to scavenge residual oxygen. 10 m*M* dithiothreitol and 100 µ*M* ubiquinone-1 were used as an electron-donation system. Inhibition-assay activity measurements were performed by repeating the above experiments with the addition of 150 µ*M* divalent metal ions (MgCl_2_, CaCl_2_, ZnCl_2_, CdCl_2_ and ZnSO_4_) to the assay mixture (Supplementary Table S2). *Ax*qNOR Glu494Ala-BRIL activity was measured in a similar fashion as previously reported (Gopalasingam *et al.*, 2019 ▸). NO-reduction rates were calculated using the *Igor Pro* software package (https://www.wavemetrics.com/).

### 2.4. Protein crystallization, data collection and structure determination   

*Nm*qNOR crystals were grown from peak 1 chromatographic fractions using the hanging-drop vapour-diffusion method, with protein at a concentration of 10 mg ml^−1^ and a reservoir solution consisting of 50 m*M* HEPES pH 8, 26%(*v*/*v*) PEG 600, 0.5 m*M* ZnSO_4_. Crystals were cryoprotected in 20% glycerol and subsequently flash-cooled and stored in liquid nitrogen. Diffraction data were obtained to 3.0 Å resolution on the BL44XU beamline at SPring-8, Japan at 100 K using a CCD detector. The data were processed by *iMosflm* (Battye *et al.*, 2011 ▸) and then scaled using *AIMLESS* (Evans & Murshudov, 2013 ▸). The structure was solved by molecular replacement with *MOLREP* (Vagin & Teplyakov, 2010 ▸) using the cryo-EM structure of wild-type *Ax*qNOR as a starting model (PDB entry 6qq5; Gopalasingam *et al.*, 2019 ▸). The structure was refined by *REFMAC*5 (Murshudov *et al.*, 2011 ▸) and manually rebuilt within *Coot* (Emsley *et al.*, 2010 ▸). Data-collection and refinement statistics are included in Supplementary Table S1.

### 2.5. Cryo-EM sample vitrification and data acquisition   

Prior to cryo-EM screening, negative-stain screening was performed to confirm the suitability of the sample for cryo-EM analysis. For cryo-EM data collection, 3 µl *Nm*qNOR-BRIL (peak 1) at 4 mg ml^−1^ was applied onto glow-discharged (Pelco) R1.2/R1.3 Cu holey carbon grids (Quantifoil). The grids were blotted using a blot force of 6 for 6 s and were then plunge-frozen into liquid ethane using a Vitrobot Mark IV (Thermo Fisher Scientific) maintained at 4°C with ∼98% humidity. Screening and data collection took place at the Astbury Bio­Structure Laboratory (University of Leeds, UK) using a Titan Krios (Thermo Fisher Scientific) operating at an acceleration voltage of 300 kV and equipped with a K2 Summit detector (Gatan) in counting mode. Imaging was performed at a nominal magnification of 130 000×, leading to a calibrated pixel size of 1.07 Å per pixel. A total of 3182 movies were recorded. The dose rate was 8.68 e^−^ Å^−2^ s^−1^ recorded over 8 s, captured in 40 frames, resulting in a total dose of 69.44 e^−^ Å^−2^, with a defocus range from −1 to −3.5 µm. Data collection was performed using *EPU* (Thermo Fisher Scientific). *Ax*qNOR Glu494Ala-BRIL grids were prepared in a similar fashion to those for *Nm*qNOR-BRIL, except that a 3 mg ml^−1^ sample from peak 2 was used. Data were collected at the electron Bio-Imaging Centre, Diamond Light Source, UK using a Titan Krios (microscope M03) operating at 300 kV equipped with a K2 Summit detector in counting mode. Imaging was performed at a nominal magnification of 48 000×, leading to a calibrated pixel size of 1.043 Å per pixel. A total of 2239 movies were recorded with a dose rate of 4.66 e^−^ Å^−2^ s^−1^ recorded over 12 s, captured over 40 frames, resulting in a total dose of 55.9 e^−^ Å^−2^, with a defocus range from −1.5 to −3 µm.

### 2.6. Image processing of cryo-EM movies   

#### 2.6.1. *Nm*qNOR-BRIL (peak 1 fraction)   

All image processing was performed in *RELION*-3.0 (Zivanov *et al.*, 2018 ▸) unless stated otherwise. Movies were motion-corrected (with dose-weighting) using the *RELION*-3.0 implementation of *MotionCor*. CTF estimation was performed using *CTFFIND* 4.1.10 (Rohou & Grigorieff, 2015 ▸). An initial 2100 particles were manually picked to generate templates for auto-picking of all micrographs. After auto-picking, ∼970 000 particles were extracted (200-pixel box size), which were then subjected to particle sorting to eliminate ‘junk’ particles. After sorting, ∼800 000 particles were subjected to 2D classification (four rounds with 7 Å expectation step limit) which generated classes with clear dimeric particles and elements of secondary structure. An initial model was generated (*C*1 symmetry) which was then used during 3D classification (*K* = 4, 10 Å expectation step limit) upon filtering to 50 Å. The best classes from each round of classification served as references for the next round. After four rounds of 3D classification with the previous settings, two rounds (*K* = 3, no expectation step resolution limit) of classification left the best class composed of 233 556 particles. 3D auto-refinement of this class led to a resolution of 4.2 Å in *C*1 symmetry, with map sharpening yielding a 3.9 Å resolution reconstruction. Refinement in *C*2 symmetry led to a resolution of 3.7 Å, with map sharpening using a tight solvent mask (excluding the detergent belt and BRIL molecules, with 6 pixels cosine edge added) improving the resolution to 3.4 Å. These particles were then subjected to CTF refinement, with CTF parameter fitting and re-estimation of per-particle defocus values. Refinement and map sharpening led to a map of 3.5 Å resolution, before Bayesian particle polishing was performed using all movie frames and parameters previously determined from a training step which used 25 000 particles. The ‘shiny’ particles were auto-refined to 3.3 Å resolution, with map sharpening using a tight mask leading to a final resolution of 3.06 Å. All resolution estimates were calculated using the ‘gold standard’ Fourier shell correlation (FSC) = 0.143 (Scheres & Chen, 2012 ▸). *ResMap* version 1.95 (Kucukelbir *et al.*, 2014 ▸) was used to assess slices through local resolution estimates, using the default finest and coarsest resolution limits (2× and 4× pixel size, respectively). Map volumes and masks were visualized in *UCSF Chimera* version 1.13.1 (Pettersen *et al.*, 2004 ▸).

#### 2.6.2. *Ax*qNOR Glu494Ala-BRIL (peak 2 fraction)   

The *Ax*qNOR Glu494Ala-BRIL peak 2 (∼85 kDa) data were processed in a similar fashion to those for *Nm*qNOR-BRIL, except for the 3D classification steps. After several rounds of 2D classification (box size 150 pixels), an initial model was generated (*C*1 symmetry) that clearly showed a monomeric structure. 3D classification was performed using a regularization parameter (*T*) of 8 as opposed to the default of 4, to increase the weighting towards the experimental data, as performed during image processing of sub-100 kDa protein complexes (Herzik *et al.*, 2019 ▸). Three rounds of classification (*K* = 3, expectation step limit of 8 Å) yielded two classes comprising ∼150 000 particles (from an initial particle set of 680 000). Auto refinement led to a global resolution of 5 Å. CTF refinement (per-particle defocus value re-estimation) and Bayesian particle polishing followed before a final round of 2D classification, leaving ∼144 000 polished particles. Auto refinement led to a resolution of 5.05 Å and refinement was continued with a soft mask (encompassing the BRIL and detergent micelle), improving the resolution to 4.88 Å. Map sharpening with a tight mask, which excluded BRIL (with a soft cosine edge of 6 pixels), led to a final map at 4.5 Å resolution (using ‘gold standard’ FSC = 0.143), with the local resolution of the core valued at ∼4.2 Å (as judged by *RELION*-3.0).

### 2.7. Model building and refinement of cryo-EM structures   

#### 2.7.1. *Nm*qNOR-BRIL   

The X-ray model of *Nm*qNOR (from this work) was rigidly docked into the final cryo-EM map using the *UCSF Chimera* ‘Fit in Map’ function before visualization in *Coot*. In parallel, the *ARP*/*wARP* (Langer *et al.*, 2008 ▸) EM webserver (https://arpwarp.embl-hamburg.de/) was also used to build the model *ab initio*. *ARP*/*wARP* correctly identified and positioned 96% of the residues. When manual model building took place, the missing loop regions on the cytoplasmic side and modified helices were taken from the *ARP*/*wARP* EM webserver model. Once built, the model was real-space refined using the *phenix.real_space_refine* module in *Phenix* version 1.15 (Afonine *et al.*, 2018 ▸), imposing secondary-structure and noncrystallographic symmetry (NCS) restraints. Model morphing was only performed once at the start of each refinement. After several rounds of manual building in *Coot* and refinement, validation of the model was performed using *MolProbity* (Williams *et al.*, 2018 ▸). Cross-validation of the refined model was performed to test for overfitting (Brown *et al.*, 2015 ▸). The refined model was ‘shaken’ by inducing random 0.5 Å shifts in the atomic co­ordinates, which was performed in *phenix.pdbtools* within *Phenix*. The shaken model was then refined against the first half map. The shaken-refined model was used to calculate the FSC against the same half map as used in refinement (FSC_work_), against the second half map that was not used in refinement (FSC_free_) and finally against the summed map (FSC_sum_). These were performed in *phenix.mtriage* within *Phenix*. The minor difference between the FSC_work_ and FSC_free_ curves indicated no major overfitting of the model into the maps.

#### 2.7.2. *Ax*qNOR Glu494Ala-BRIL   

The *Ax*qNOR Glu494Ala structure was refined with *phenix.real_space_refine* in *Phenix* version 1.15 without the nonheme Fe. The Glu490 and His486 side chains were also removed from the model as no clear density was observed. Secondary-structure, Ramachandran and rotamer restraints were applied during refinement, with model morphing performed once at the start of refinement. Owing to the lower resolution of the density map, tighter geometry restraints were employed in the early rounds of refinement by lowering the weighting term from the default of 50 to 25, which greatly reduced the all-atom clashscore and improved the overall geometry. Further improvements were made by employing reference-based restraints using the *Ax*qNOR Val495Ala structure (PDB entry 6qq6; Gopalasingam *et al.*, 2019 ▸) as a reference model. *Coot* was used to manually rebuild the model, with validation performed using *MolProbity*. As with all qNOR-BRIL cryo-EM structures thus far, the BRIL portion was not built into the model owing to a lack of clear, detailed density as a result of its flexibility. Cross-validation of the refined model was performed in the same manner as for *Nm*qNOR-BRIL.

## 3. Results   

### 3.1. Oligomerization state of *Nm*qNOR   

**Figure 1:**
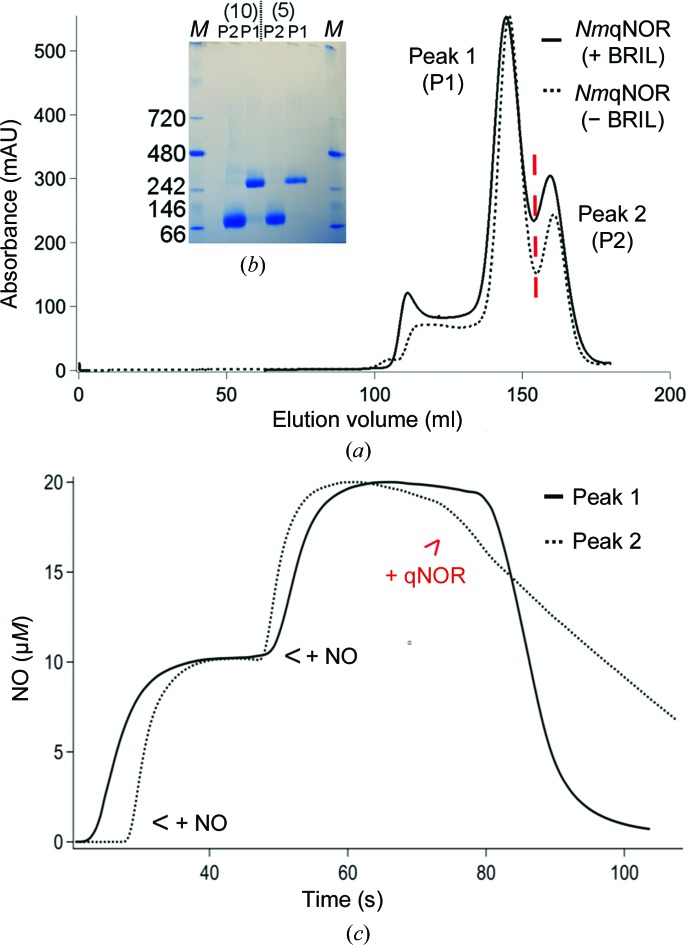
Biochemical characterization of *Nm*qNOR. (*a*) Size-exclusion chromatography of *Nm*qNOR. The solid curve shows the BRIL construct of *Nm*qNOR and the broken curve shows the wild-type *Nm*qNOR trace at 280 nm absorbance. Two types of fractions were observed in both constructs, which are indicated by the labels P1 for dimer and P2 for monomer (the red dashed line shows the divide between peak 1 and peak 2). (*b*) Blue native gel experiment for both fractions (P1 and P2) after concentration in 100 kDa cutoff centrifugal filtration devices (Amicon). Both 5 µg (labelled 5) and 10 µg (labelled 10) of each fraction was loaded onto the gel. Peak 1 samples migrate as the higher molecular-weight species (∼242 kDa), whilst peak 2 samples migrate between 66 and 146 kDa. Two bands of the high and low molecular-weight species could be observed in both peak fractions when loaded with 10 µg sample, which indicate a slight mixture of monomer and dimer in both peak 1 and peak 2. Lane *M*, native PAGE marker, with values corresponding to molecular weight in kDa. (*c*) NO-reduction activity measurement of both peak 1 and peak 2 fractions. Peak 1 (solid curve) shows a higher NO-reduction activity compared with peak 2 (broken curve).

During size-exclusion chromatography, *Nm*qNOR displays a dimer–monomer mixture upon elution, with the majority of the protein in the peak 1 fraction [Fig. 1 ▸(*a*)]. This behaviour is not owing to the detergent exchange from DDM to DTM, as the migration is essentially identical. To assess the oligomeric state, we subjected the peak 1 and peak 2 fractions to non­denaturing gel electrophoresis (blue native PAGE). Pre­dominantly, peak 1 migrated to ∼242 kDa [consistent with dimeric *Nm*qNOR (90 kDa × 2) plus the detergent micelle (∼60 kDa)], whilst peak 2 migrated between 66 and 146 kDa. The gel indicated that both peaks had a slight mixed species behaviour when the sample concentration was increased [Fig. 1 ▸(*b*)]. NO reduction of each peak was conducted, with peak 1 exhibiting a higher steady-state NO-reduction activity compared with the peak 2 sample [Fig. 1 ▸(*c*)]. This is similar to *Ax*qNOR, but contrasts with bovine cytochrome *c* oxidase (C*c*O), where the activity of the monomeric species was found to be twice that of the dimeric form. The latter of these had previously been observed in crystal structures, until recently, when a monomeric bovine C*c*O structure was reported (Shinzawa-Itoh *et al.*, 2019 ▸). Metal-content measurement was performed after purification for both peak fractions of *Nm*qNOR, showing that three Fe atoms and no zinc were present in the sample solution. Nonheme iron is present in both peaks at full occupancy. UV–visible spectra (oxidized and reduced) shows similar band positions from both peak fractions, indicating electronic equivalence of the active site for both peaks [Supplementary Figs. S1(*a*) and S1(*b*)].

### 3.2. Crystal structure of *Nm*qNOR   

**Figure 2:**
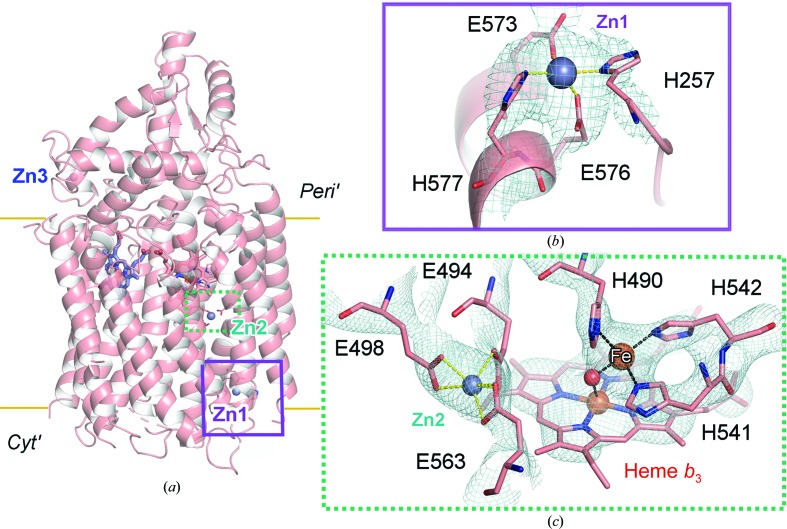
Monomeric, zinc-bound crystallographic structure of *Nm*qNOR. (*a*) Monomeric *Nm*qNOR crystal structure with three zinc-binding sites (grey spheres; Zn1, Zn2 and Zn3) and with the periplasmic (*Peri′*) and cytoplasmic (*Cyt′*) ends of the lipid bilayer marked with yellow lines. (*b*) The Zn1 binding site near the proposed proton-entry site at the cytoplasmic end; the 2*F*_o_ − *F*_c_ density map is shown as a blue mesh, with Zn1 ligated by His257 (contoured at 1.9σ), Glu573 (contoured at 2.4σ), Glu576 (contoured at 3σ) and His577 (contoured at 2σ) (salmon sticks), (*c*) Zn2 is located close to the binuclear centre (heme *b*_3_ and Fe_*B*_; brown sphere), where it is bound by three conserved glutamates [Glu498 (1.5σ), Glu494 (1σ) and Glu563 (1σ)]. Fe_*B*_ is ligated by only three histidines (His490, His541 and His542; all contoured at 1.5σ), with extra density between the heme *b*_3_ iron and nonheme iron modelled as a water (red sphere).

The best, highly diffracting crystals of *Nm*qNOR only grew in the presence of zinc sulfate, which was used in the crystallization condition. The crystals diffracted to ∼3 Å resolution, leading to a refined structure at 3.15 Å resolution in space group *P*2_1_2_1_2_1_ (Supplementary Table S1), revealing only a monomer in the crystal lattice. The crystallization condition was similar for a bacterial C*c*O, where higher resolution crystals were only obtained in the presence of divalent metal cations (Qin *et al.*, 2007 ▸). The overall monomeric structure of *Nm*qNOR contains 14 transmembrane helices (TMs) and the positions of heme *b*, heme *b*_3_ and Fe_*B*_ are well defined [Fig. 2 ▸(*a*)] (Gonska *et al.*, 2018 ▸). Fe_*B*_ was ligated by three conserved histidines (His490, His541 and His542). Additionally, three Zn^2+^-binding sites were observed, of which the Zn1 and Zn2 binding locations were particularly important in order to understand the properties of the proton-transfer pathway. Zn1 is positioned near TMII, close to the predicted entry to the water channel, and is ligated by His257, Glu573, Glu576 and His577 [Fig. 2 ▸(*b*)]. The Zn2 ion was bound by three carboxylate groups from Glu494, Glu498 and Glu563, which are located towards the end of the putative proton-transfer water channel, in which Glu494 is too far away for coordination of Fe_*B*_ [Fig. 2 ▸(*c*)]. The third binding site for Zn (Zn3) was observed near the periplasmic region where the zinc ion has three waters bound, in addition to His172, which itself is ligated to Glu709.

### 3.3. Inhibition of NO reduction by metal ions   

In view of the zinc binding in the crystallographic structure of *Nm*qNOR, we tested the effect of several divalent metals, including zinc, on NO reduction of both *Nm*qNOR and *Ax*qNOR (Supplementary Table S2). The presence of zinc totally abolished the NO-reductase activity for both enzymes, while the presence of other divalent cations, such as magnesium and calcium, did not significantly reduce the activity. These results are largely comparable to that of C*c*O, in that both zinc and cadmium are potent inhibitors of activity (Qin *et al.*, 2007 ▸), suggesting that qNORs also suffer from proton-transfer inhibition owing to zinc/cadmium binding.

### 3.4. Cryo-EM of *Nm*qNOR reveals a dimeric structure   

**Figure 3:**
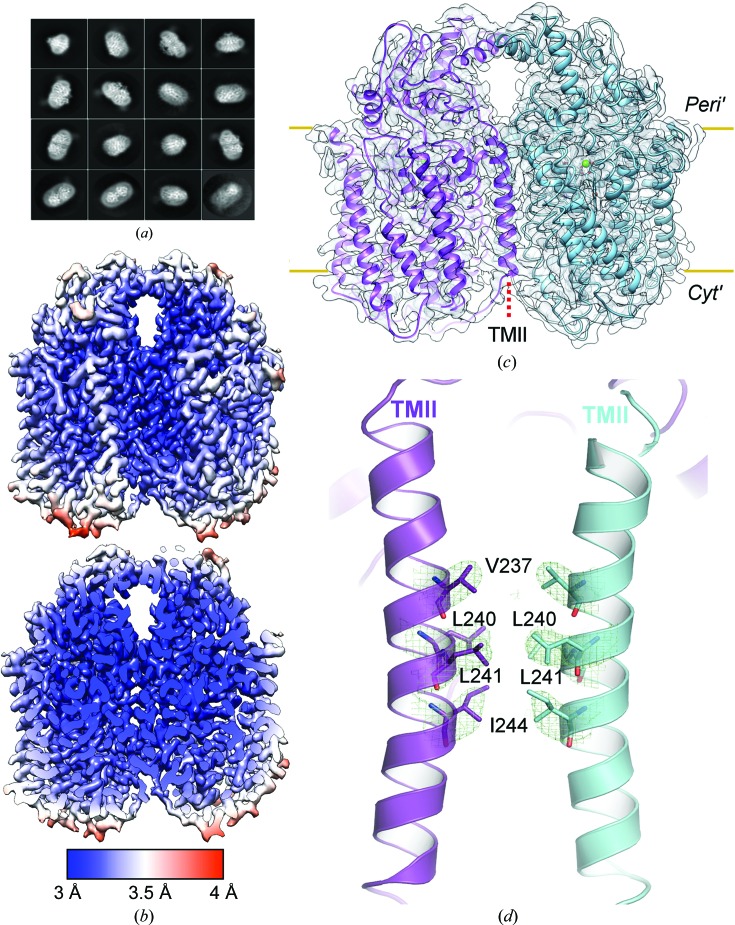
Cryo-EM analysis of the active *Nm*qNOR dimer. (*a*) 2D class averages of dimeric *Nm*qNOR particles in multiple orientations after particle polishing, prior to the final 3D refinement (box size of 200 pixels). (*b*) 3D reconstruction from cryo-EM filtered and coloured by local resolution (as estimated in *RELION*-3.0), with the core of the structure being valued at ∼3 Å (as indicated by the colour key at the bottom). (*c*) *Nm*qNOR dimeric model after structure refinement and model building docked into the sharpened cryo-EM-derived map (grey outline). Chains are coloured as purple and cyan cartoons, respectively. Yellow lines mark the lipid bilayer and subsequently the periplasmic (*Peri′*) and cytoplasmic (*Cyt′*) sides. The red dashed line indicates the location of TMII. (*d*) The dimer interface of *Nm*qNOR is mediated by TMII (purple and cyan helices) of each monomer. Val237, Leu240, Leu241 and Ile244 (map contoured at 7σ) are likely to stabilize the dimeric form of *Nm*qNOR.

Given the monomeric crystallographic structure of *Nm*qNOR, apocytochrome *b*_562_ (BRIL; Chu *et al.*, 2002 ▸) was fused to the nontruncated C-terminus of *Nm*qNOR in order to increase the molecular weight to make cryo-EM analysis tractable [Supplementary Fig. S2(*a*)]. Previous attempts to obtain a reconstruction in the absence of BRIL resulted in an ∼9 Å resolution map with a lack of clear secondary-structure features, as reported in Gopalasingam *et al.* (2019 ▸). The NO-reduction activity of *Nm*qNOR-BRIL showed no significant difference compared with that of wild-type *Nm*qNOR. After negative-staining imaging to confirm the homogeneity and integrity of the sample, grids were prepared for cryo-EM analysis. From 3182 micrographs, 972 078 initial particles were extracted, before being reduced to 233 556 particles through iterative cycles of 2D and 3D classification [Fig. 3 ▸(*a*)]. For the final 3D reconstruction with *C*2 symmetry imposed, a final global resolution of 3.06 Å was achieved, as judged by the ‘gold standard’ FSC = 0.143 (Supplementary Table S2 and Supplementary Fig. S3). The density for the BRIL portion was at a lower resolution compared with the *Nm*qNOR molecule, presumably owing to the flexibility of BRIL, and was subsequently not built into the model [Fig. 3 ▸(*b*), Supplementary Fig. S2(*c*)]. Unlike in the crystal structure [Fig. 2 ▸(*a*)], the peak 1 fraction used for cryo-EM revealed *Nm*qNOR to be in a dimeric arrangement, akin to the cryo-EM structure of *Ax*qNOR [Fig. 3 ▸(*c*)]. This dimeric structure is maintained by TMII of both monomers, with four amino-acid residues maintaining the interface: Val237, Leu240, Leu241 and Ile244 [Fig. 3 ▸(*d*)]. These four residues are highly conserved amongst most qNORs (Supplementary Fig. S4), in which they are likely to play significant roles in qNOR dimerization.

**Figure 4:**
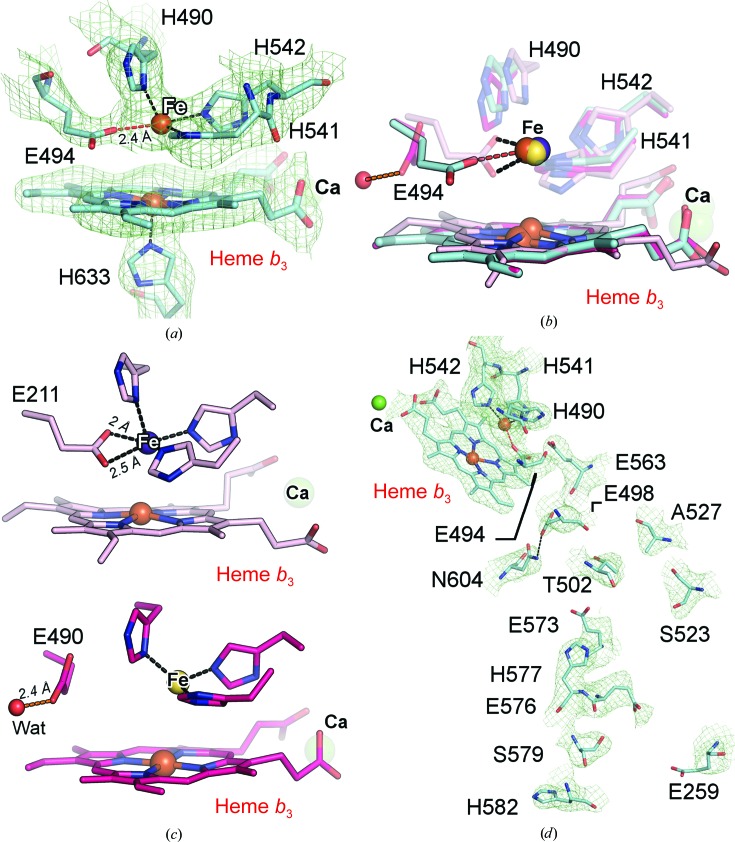
Active-site configuration and proton-transfer channel in the dimeric cryo-EM structure of *Nm*qNOR. (*a*) Active site of the ‘native’ *Nm*qNOR cryo-EM structure with the corresponding density, showing Fe_*B*_ (brown sphere) to be ligated by four ligands (Glu494, His490, His541 and His542), with Glu494 contoured at 4σ and the histidines at 8σ. The Glu494–Fe_*B*_ bond is shown as a red dashed line (2.4 Å) and the trihistidyl–Fe_*B*_ coordination is shown as black dashed lines. (*b*) Comparison between the active-site structures of *Pa*cNOR (transparent pink sticks), *Ax*qNOR V495A (transparent magenta sticks) and *Nm*qNOR (cyan sticks), clearly showing that the terminal glutamate of *Pa*cNOR and *Nm*qNOR (Glu494) ligates Fe_*B*_ (black and red dashed lines, respectively). Glu494 may display flexibility as part of its function in proton transfer, as the equivalent glutamate in *Ax*qNOR ligates a water (orange sphere) in lieu of Fe_*B*_. (*c*) Top: active site of *Pa*cNOR (PDB entry 3o0r; Hino *et al.*, 2010 ▸), with Glu211 (equivalent to Glu494 in *Nm*qNOR) ligating Fe_*B*_ (purple sphere) in a bidentate fashion, as shown by the black dashed lines (distances are also shown). Bottom: active site of *Ax*qNOR V495A (PDB entry 6qq6) showing Glu490 (equivalent to Glu494 in *Nm*qNOR) ligating a water molecule (Wat; orange sphere), with Fe_*B*_ (yellow sphere) having trihistidyl coordination. (*d*) Putative proton-transfer channel in *Nm*qNOR, with residues shown as cyan sticks and contoured at 6σ (except for Glu259 and Glu573, which are both contoured at 4σ). Asn604 and Glu498 are hydrogen-bonded (black dashed line) in the cryo-EM structure; Fe_*B*_ and calcium ions are shown as brown and green spheres, respectively.

The cryo-EM density map was more detailed than the crystallographic electron-density maps, in which heme *b*, heme *b*_3_, Fe_*B*_ and their associated ligands could be modelled with confidence. The coordination of the active site in the absence of zinc was observed by cryo-EM, which interestingly showed that Fe_*B*_ was ligated by four ligands: the three conserved histidine ligands (His490, His541 and His542) and Glu494 [Fig. 4 ▸(*a*)]. The ligation mode of Glu494 is different from that in the previously reported (inactive) crystal structure of *Geobacillus stearothermophilus* qNOR (*Gs*qNOR) and in the active *Ax*qNOR structure obtained by cryo-EM, both of which showed trihistidyl ligand coordination of Fe_*B*_ (Matsumoto *et al.*, 2012 ▸; Gopalasingam *et al.*, 2019 ▸) [Fig. 4 ▸(*b*)]. The orientation of this conserved glutamate in the *Nm*qNOR cryo-EM structure is similar to that of *Pseudomonas aeruginosa* cNOR (*Pa*cNOR; Hino *et al.*, 2010 ▸), except that in *Pa*cNOR Fe_*B*_ was coordinated by both carboxyl O atoms of the glutamate (at distances of 2 and 2.5 Å), while in the *Nm*qNOR cryo-EM structure Fe_*B*_ is coordinated by a single carboxyl O atom (O^∊1^) from Glu494, separated by 2.4 Å [Fig. 4 ▸(*c*)]. Another difference in the active sites of the *Nm*qNOR EM structure and the cryo-EM structure of *Ax*qNOR is the distance between Fe_*B*_ and the heme *b*_3_ iron, which is 4.5 Å in this structure and 4.1 Å in *Ax*qNOR. These differences in the active-site structure may reflect a significant difference in NO-reduction activity for the two qNOR enzymes (Supplementary Table S2).

This higher quality of the resultant structure enabled us to investigate the details of the putative proton-transfer water channel in this electrogenic enzyme. A large cavity was observed from the cytoplasmic region near residues Ser252, Thr255, Glu259, Tyr575, Glu576 and Ser579 towards the active site [Supplementary Fig. S5(*a*)]. This helps to rationalize our previous site-directed mutagenesis study of the proposed water-entry channel, in which Glu259 variants had no detrimental effect on *Nm*qNOR activity, as the channel may be too large, allowing some potential redundancy in proton pathway(s) (Gonska *et al.*, 2018 ▸).

The dimeric structure of *Nm*qNOR is largely maintained by TMII, similar to as in *Ax*qNOR. Along TMII, structural analysis using *PyMOL* (version 1.8; Schrödinger) revealed a cavity that bridges the dimeric interface connecting the two monomers [Supplementary Figs. S5(*b*) and S5(*c*)]. There are several residues which maintain this cavity: Tyr251, Ser252, Thr255 and Arg572. These in turn connect with the charged residues that line the putative proton-transfer water pathways of the respective monomers. This pathway consists of several charged and ionizable residues, starting from Glu259, Ser579 and His582, and followed by Glu573, His577, Glu576, Ser523, Thr502 and Ala527 [Fig. 4 ▸(*d*)]. At the end of this putative water channel lie Glu498 and Asn604, which are ligated and may serve as a ‘junction’ that is responsible for controlling the proton before entering the active site; our previous study shows that mutation of Glu498 reduces the activity of this enzyme (Gonska *et al.*, 2018 ▸). The same effect was found in *Ax*qNOR, with Asn600 (equivalent to Asn604 in *Nm*qNOR) being critical for activity [as also confirmed in *Persephonella marina* qNOR (*Pm*qNOR; Sheraden, 2013 ▸)], with residues closer to the active site of *Nm*qNOR and *Ax*qNOR being more conserved in residue type and orientation compared with those lower down (*i.e.* towards the cytoplasmic end of) the putative proton-transfer water channel (Supplementary Fig. S6 and Supplementary Table S4).

### 3.5. The cryo-EM structure of monomeric *Ax*qNOR Glu494Ala provides insight into the dimer–monomer transition of qNORs   

**Figure 5:**
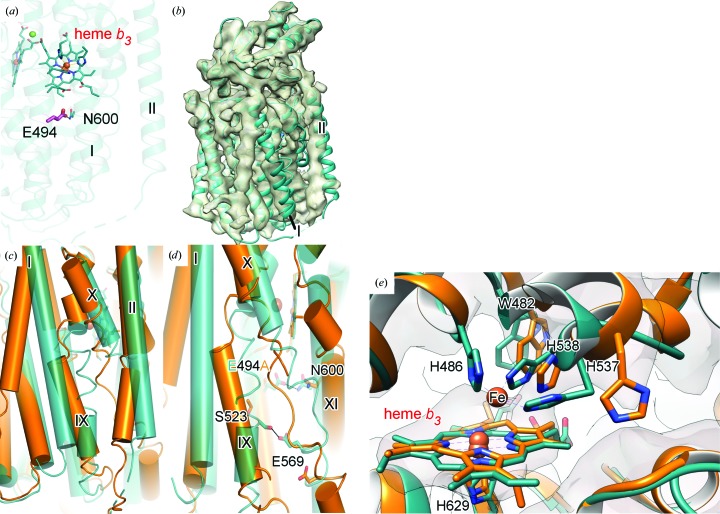
Proton-transfer inhibition in a monomer of *Ax*qNOR Glu494Ala. (*a*) The location of Glu494 (magenta sticks) in wild-type *Ax*qNOR (teal cartoon; PDB entry 6qq5) relative to the dimer-stabilizing TMII. (*b*) 4.5 Å resolution *Ax*qNOR Glu494Ala peak 2 cryo-EM-derived density map (khaki) with wild-type *Ax*qNOR (teal cartoon) rigidly docked into the map, showing displacement of TMI and TMII. (*c*) Alignment of wild-type *Ax*qNOR (teal cylinders) and the *Ax*qNOR Glu494Ala peak 2 structure (orange cylinders), with an overall r.m.s.d. of 1.06 Å, with the helices showing the greatest displacement, TMI, TMII, TMIX and TMX, labelled with roman numerals. (*d*) Disruption of the putative proton-transfer channel by movement of TMX of the *Ax*qNOR Glu494Ala peak 2 structure (orange) across the cavity (∼8 Å from Ile564C^α^), with the Glu569–Ser523 interaction in the wild-type structure (teal) likely to be perturbed as a result. Asn600 shows a conserved conformation, despite being hydrogen-bonded to Glu494 in the wild-type structure. (*e*) Binuclear centre of the *Ax*qNOR Glu494Ala monomer (orange cartoon, grey cryo-EM density map contoured at 3σ) compared with wild-type *Ax*qNOR (teal cartoon), with no density for His486 (omitted from the *Ax*qNOR Glu494Ala peak 2 model) and unclear density for the nonheme metal. The His537 and His538 conformations are significantly altered in the absence of the nonheme metal and could contribute to downstream structural movements.

Glu498 variants in *Nm*qNOR resulted in a loss of activity of greater than 90%, with the same effect holding true for the corresponding residue in *Ax*qNOR (Glu494). This residue is fully conserved amongst all NORs and is believed to partake in proton transfer towards the active site in qNORs, being hydrogen-bonded to Asn600 in *Ax*qNOR (Asn604 in *Nm*qNOR) [Fig. 5 ▸(*a*)], in the aforementioned ‘junction’ before the active site. The *Ax*qNOR Glu494Ala-BRIL variant purified largely as a monomeric species [Supplementary Fig. S7(*a*)], in contrast to the purification profiles of the wild type and other mutant enzymes. The Glu494Ala monomeric species was inactive, while the dimeric species showed a detectable NO-reduction activity of less than 10% [Supplementary Fig. S7(*b*)]. The predominance of the monomeric peak for this mutant provided an opportunity to determine the cryo-EM structure of the monomeric species in order to help to ascertain the structural elements that may play a role in the dimer–monomer transition of qNORs.

The structure of the ∼85 kDa monomer was determined to ∼4.5 Å resolution [Supplementary Table S3, Supplementary Figs. S7(*c*)–S7(*e*)] by cryo-EM. The BRIL portion could not be built owing to its greater flexibility and limited resolution [Supplementary Figs. S2(*b*) and S2(*d*)]. The structure revealed large movements of TMI (∼10 Å displacement of the Val17 C^α^ atom) and the dimer-stabilizing TMII helix [Fig. 5 ▸(*b*)] relative to the wild-type enzyme structure [Fig. 5 ▸(*c*)]. TMX was also shifted across and into the putative proton-transfer channel (∼8 Å movement of the Ile564 C^α^ atom), acting to potentially perturb the channel [Fig. 5 ▸(*d*)]. TMX contains Glu569, which was found to interact with Ser523 (on TMIX) in the wild-type structure, with the substitution of the latter causing a 70% loss of activity, believed to be owing to the loss of the Glu569 interaction.

This is likely to be disrupted as a consequence of the Glu494Ala point mutation. Additionally, the binuclear centre was perturbed, with no density for His486 and a weak density at the position expected for the nonheme iron (this iron was therefore removed from the model) [Fig. 5 ▸(*e*)]. TMIX, which contains two of the three Fe_*B*_ ligands, His537 and His538, is very distorted. The loss of iron may itself result in these conformational changes or they may induce a weakening of the iron-binding site. Regardless, they cause more pronounced changes of the dimer-stabilization and proton channel-flanking helices, confirming Glu494 to be a critical residue not only for activity but also for structural maintenance of the dimer and active site in *Ax*qNOR.

## 4. Discussion   

### 4.1. Structural comparison of the crystallographic monomer and the cryo-EM dimer of *Nm*qNOR   

**Figure 6:**
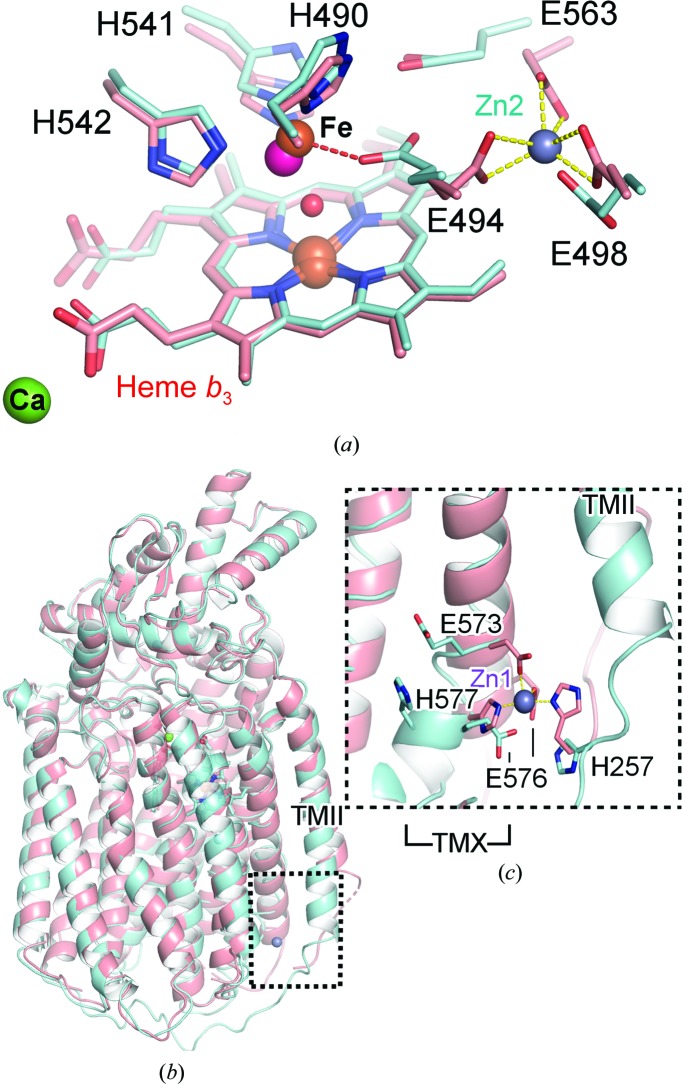
Comparison of dimeric cryo-EM and monomeric crystallographic *Nm*qNOR structures. (*a*) In the crystallographic structure (salmon sticks), the Zn2 binding site (grey sphere) interferes with Fe_*B*_ (magenta sphere), as Glu494 is helping to coordinate Zn2 (yellow dashed lines), whilst the native, non-zinc-bound cryo-EM-derived structure (cyan sticks) shows Glu494 to ligate Fe_*B*_ (brown sphere) in a monodentate fashion via the O^∊1^ atom, as shown by the red dashed line. (*b*) Alignment of the crystallographic (salmon cartoon) and cryo-EM structures (one protomer of the dimer is shown as a cyan cartoon) shows distortion of TMII in the crystal structure, possibly owing to the absence of a neighbouring molecule. TMX is also heavily perturbed, possibly owing to Zn1 binding [black dashed box, elaborated in (*c*)]. (*c*) Zn1 binding causes significant helical movement of TMX, with the cryo-EM TMX shifted inwards, with Glu573, Glu576 and His577 facing towards the putative proton-transfer channel/solvent in the absence of zinc, with His257 facing towards the cytoplasmic end.

Based on our results, the crystal and cryo-EM structures of *Nm*qNOR have different active-site arrangements. For the crystal structure, the crystal was grown under zinc-containing conditions, which led to the observation of three zinc-binding sites, with the Zn2 binding site affecting the conformation of Glu494. However, a native dimeric structure was observed in the cryo-EM structure as the data were collected without any additional buffer components; in this structure Glu494 was ligated to Fe_*B*_, which we believe is the natural position for Glu494 [Fig. 6 ▸(*a*)]. However, knowing that the inhibitor (zinc) binds toward the end of putative water channel [Fig. 2 ▸(*c*)] in the crystal structure, Glu494 may possess some flexibility between Fe_*B*_ and the end of the putative proton-transfer pathway, as seen in *Ax*qNOR (Gopalasingam *et al.*, 2019 ▸). From the overall structure, TMII in the crystal structure moves significantly outwards compared with the cryo-EM structure. The TMII helix is clearly important for maintaining the dimer interface, and a distortion of this helix may be a driver for disruption of the active dimer [Fig. 6 ▸(*b*)].

Another possible reason was the location of Zn1 binding in the early part of the putative proton-transfer pathway. Three amino acids which bind Zn1 may be responsible for the putative proton-transfer pathway and cavity formation/maintenance, as observed in the dimeric cryo-EM structure and shown in Supplementary Fig. S5(*c*). This binding of Zn1 might be responsible for the change in cavity structure and also the loss of activity in the presence of zinc, and affect the position of TMII in the crystal structure. The position of His577 in the cryo-EM structure was moved upwards and Glu576 and Glu573 were moved downwards in order to accommodate space for the potential water pathway in the cavity; however, in the crystal structure all three of these residues bind atom Zn1 [Fig. 6 ▸(*c*)]. Alignment of the monomer and dimer structures of *Nm*qNOR shows that Zn1 binding may cause the TMII loop region to splay away and into the symmetry-related molecule of the dimer, specifically clashing with TMX (Supplementary Fig. S8). This structural change may be an important factor in the dissolution of the dimer *in crystallo*.

### 4.2. Active-site structure of *Nm*qNOR   

Based on the structure of active *Ax*qNOR and the inactive crystal structure of *Gs*qNOR, it has been suggested that Fe_*B*_ of qNOR is ligated by only three conserved histidine ligands. The cryo-EM structure of *Nm*qNOR, like *Ax*qNOR, is dimeric; however, Fe_*B*_ has four ligands. The activity of *Nm*qNOR is an order of magnitude higher than that of *Ax*qNOR, which may arise from this difference in the ligand environment of the active site. We suggest that Glu494 (*Nm*qNOR numbering) plays a vital role as a final proton acceptor before transferring the proton to the active site for NO reduction. The flexible nature of this catalytically essential Glu494 might be unique to qNOR and a key property for fulfilling its function of proton transfer from either Glu498 or Glu563. The result of zinc binding in the Zn2 site shows that these three main glutamates (Glu498, Glu494 and Glu563) were grouped together by zinc binding, in which Glu494 can receive protons either directly from Glu498 or through Glu563. Whether the flexibility of glutamate facilitates the accommodation of two NO molecules for NO reduction in the active site remains an open question.

### 4.3. Putative proton-transfer water channel   

Zn^2+^ and Cd^2+^ ions have been utilized in several membrane-protein complexes in order to understand their respective proton-transfer pathways. These include bacterial and bovine C*c*O, the cytochrome *bc*_1_ complex and the photosynthetic light reaction centre (Schubert *et al.*, 1997 ▸; Berry *et al.*, 2000 ▸; Muramoto *et al.*, 2007 ▸; Qin *et al.*, 2007 ▸). Zinc binds towards the D-pathway in bovine C*c*O and the K-pathway in bacterial C*c*O; thus, as a part of the HCuO family, the properties of Zn and Cd in binding either towards the imidazole ring of histidine and three carboxyl groups has proven to be very useful in order to understand the properties of the proton pathway in *Nm*qNOR. The Zn1 and Zn2 binding sites were crucial in this study, as we predict that the Zn1 binding site is at the entrance to the putative proton-transfer water channel and that the Zn2 binding site represents the end of the channel. Previously, we showed that site-directed mutagenesis around the entrance does not have any significant effect on the activity of *Nm*qNOR (Gonska *et al.*, 2018 ▸), which is consistent with the large cavity entrance towards the catalytic site. To our knowledge, qNOR is the only member of the HCuOs that has a large water cavity from the cytoplasmic end towards the active site.

The opening cavity of the water channel is formed by several residues (Ser252, Thr255, His257, Glu259 and Ser579). The cavity then moves upwards and broadens between Glu573 and Ser523. At the end of the putative proton-transfer pathway observed in the cryo-EM-derived structure, it is shown that the proton-transfer pathway could be controlled by two amino-acid residues: Glu498 and Asn604. This is further validated by the binding of zinc (in the Zn2 location) and the previously reported site-directed mutagenesis of Glu498, which has significant effects on abolishing activity. We expect that the putative water channel terminates here, and protons are transferred towards Glu563, mediated by the flexible Glu494. Alternatively, transfer of protons can occur directly from Glu498 to Glu494, ultimately reaching the active site. The monomeric, inactive structure of *Ax*qNOR Glu494Ala revealed unexpected structural rearrangements at the active site and several helices. TMII undergoes a slight displacement, while the helices TMIX and TMX, which help to maintain the putative proton-transfer channel, revealed a more drastic change [Supplementary Fig. S8(*b*)]. The disturbance at the active site probably had a knock-on effect on the overall structure of TMX (which contains His537 and His538, two of the Fe_*B*_ ligands). Interestingly, TMVIII (which contains Glu494 and His486, the third Fe_*B*_ ligand) shows little re­arrangement, along with TMXII, which contains Asn600 (previously shown to interact with Glu494). Similarly, in *Nm*qNOR movement of TMXI across and into the putative channel may weaken the interaction with the symmetry-related TMII [Supplementary Fig. S8(*a*)], possibly contributing to the dimer–monomer transition. These movements and the consequent destabilization of the TMIX and TMX helices in *Ax*qNOR Glu494Ala clearly weaken the dimeric arrangement and result in the mutant purifying primarily as a monomer.

Activity measurements of the wild-type qNORs show that the dimeric peak obtained from SEC had higher activity than the monomeric peak. Structurally, this may be owing to TMII, which resides close to the entrance of the proton-transfer pathway in the symmetry-related monomers, as was clearly observed in the cryo-EM structure. The dimeric structure may cause increased stability of qNORs in the maintenance of the proton-transfer pathway, and this was clearly seen in the distortion of TMII in the monomeric crystal structure, where the helix moves outwards and away from the rest of the molecule when compared with the cryo-EM structure [Supplementary Fig. S7(*a*)]. However, it is not clear whether this distortion occurs owing to any effect resulting from zinc binding in the Zn2 site in the proton pathway, as seen in the crystal structure. The stability of the dimeric structure may synergize the proton pathway of two monomers owing to the cavity connection mentioned previously. This raises the possibility that the proton entry is shared between two monomers and increases the proton passage by way of widening the channels in the dimeric structure. For the related C*c*O (bovine), however, the monomeric form has been found to be more active at a variety of pH values. In that case, the structure revealed the K-pathway hydrogen-bonded network of water molecules to be continuous and unrestricted, whereas in the dimeric structures (Supplementary Fig. S9) cholate interrupts this network near the dimer interface (Shinzawa-Itoh *et al.*, 2019 ▸).

## 5. Concluding remarks   

In summary, we have solved the high-resolution structure of wild-type *Nm*qNOR by cryo-EM at 3.06 Å resolution and that of inhibitor (zinc)-bound *Nm*qNOR by X-ray crystallography at 3.15 Å resolution. The overall dimeric cryo-EM structure of *Nm*qNOR is similar to the dimeric cryo-EM structure of *Ax*qNOR, which together represent the structure of active qNOR in the native state. The presence of a dimeric qNOR may serve an environmental advantage for *N. meningitidis* (and *A. xylosoxidans*, an opportunistic pathogen; Awadh *et al.*, 2017 ▸) to proliferate under host cell-produced NO as a means of both detoxifying toxic byproducts and conserving energy. The monomeric crystallographic structure of *Nm*qNOR may result from disruption of the dimer interface owing to zinc binding near the dimer-stabilizing TMII region. The role of the TMII region in stabilizing the dimer is reinforced by the monomeric cryo-EM structure of the Glu494Ala mutant of *Ax*qNOR. The single mutation results in active-site disturbance of nonheme iron ligands, culminating in displacement of the dimer-stabilizing TMII and the putative proton-transfer channel flanking helices TMIX and TMX, acting to create a catalytically inert monomeric qNOR. The monomer–dimer transition of qNORs seen in cryo-EM and crystallographic structures has wider implications for structural studies of multimeric membrane proteins.

## 6. Authors contributions   

MAMJ expressed, purified, crystallized and performed enzymatic assays with *Nmq*NOR samples. CCG expressed, purified and performed enzymatic assays for *Axq*NOR Glu494Ala- 1BRIL. MAMJ, TT and KM collected X-ray data for *Nmq*NOR crystals. MAMJ, KM and SVA refined and built the *Nmq*NOR X-ray model. RMJ and SPM made cryo-EM sample grids (for both *Nmq*NOR-BRIL and *Axq*NOR Glu494Ala-BRIL) and helped to set up data collection for *Nmq*NOR-BRIL. MAMJ, CCG and RMJ processed the cryo-EM images for *Nmq*NOR-BRIL, whilst CCG processed the *Axq*NOR Glu494Ala-BRIL micrographs. MAMJ, CCG, SVA and KM refined and built the *Nmq*NOR-BRIL cryo-EM model. CCG and SVA refined and built the *Axq*NOR Glu494Ala-BRIL cryo-EM model. TT, KM, SPM, SVA, YS and SSH conceived the study. MAMJ, CCG, TT, KM, SVA, YS and SSH prepared the manuscript. All authors read and discussed the results.

## Supplementary Material

PDB reference: Zn^2+^-bound *N. meningitidis* qNOR, X-ray crystal structure, 6l1x

PDB reference: *N. meningitidis* qNOR, cryo-EM structure, 6l3h

PDB reference: *A. xylosoxidans* qNOR, Glu494Ala mutant, cryo-EM structure, 6t6v

EMDB reference: *N. meningitidis* qNOR, EMD-0822

EMDB reference: *A. xylosoxidans* qNOR, Glu494Ala mutant, EMD-10387

Supplementary Tables and Figures. DOI: 10.1107/S2052252520003656/pw5011sup1.pdf
