# Supplementary material for: The active form of quinol-dependent nitric oxide reductase from Neisseria meningitidis is a dimer
Source: IUCrJ. 2020 Mar 21;7(Pt 3):404–15. doi: 10.1107/S2052252520003656 (PMC7201271; doi:10.1107/S2052252520003656)
Supplement: Supplementary file 1 [file m-07-00404-sup1.pdf]

IUCrJ

Volume 7 (2020)

Supporting information for article:

The active form of quinol-dependent nitric oxide reductase from *Neisseria meningitidis* is a dimer

M. Arif M. Jamali, Chai C. Gopalasingam, Rachel M. Johnson, Takehiko Tosha, Kazumasa Muramoto, Stephen P. Muench, Svetlana V. Antonyuk, Yoshitsugo Shiro and Samar S. Hasnain

Table S1 Data collection and refinement statistics of *Nm*qNOR crystal structure

	*Nm*qNOR (PDB ID:6L1X)
**Data collection**	
Space group	$P2_12_12_1$
Cell dimensions	
a, *b*, *c* (Å)	96.12 116.68 123.55
α=β=γ (°)	90
Resolution (Å)	49.87- 3.10 (3.31- 3.1) *
R_{sym} or R_{merge}[a]	0.11 (0.556)
R_{pim}	0.083 (0.42)
Mean (I / (σ)I))	6.5 (2.2)
$CC_{1/2}$	0.995 (0.207)
Completeness (%)	83.3 (84.5)
Redundancy	4.1 (4.0)
Wilson *B*-factor ($Å^2$)	60.9
**Refinement**	
Resolution (Å)	49.92 – 3.15
No. Reflections	19034
R_{work} / R_{free}[b]	0.25/0.30
No. Atoms	5655
Protein	5563
Water	1
Zn	3
Free Fe	1
Ca	1
B-factors ($Å^2$)	
Protein	102.82
Water	65.67
Zn ions	121.47
R.M.S. deviations	
Bond lengths (Å)	0.0037
Bond angles (°)	0.994
**Clash score**	18
**MolProbity score**	3
**Poor rotamers (%)**	7.5
**Ramachandran Plot**	

**Favoured (%)**	92.55
**Allowed (%)**	6.02
**Outliers (%)**	1.43

*Values in parentheses are for the highest resolution shell

[a] $R_{merge} = \sum|I_i - I_m|/\sum I_i$, where I_i is the intensity of the measured reflection and I_m is the mean intensity of all symmetry related reflections.

[b] $R_{free} = \Sigma_T||F_{obs}| - |F_{calc}||/\Sigma_T|F_{obs}|$, where T is a test data set of about 5% of the total reflections randomly chosen and set aside prior to refinement.

Table S2 *Nm*qNOR and *Ax*qNOR divalent metal inhibition activity assays

**Metal ion**	***Nm*qNOR Activity (µmol-NO/s/µmol- qNOR)**	***Ax*qNOR Activity (µmol-NO/s/µmol- qNOR)**
**No Metal**	37 ± 2	4 ± 0.5
**$MgCl_2$**	35.1± 2.4	3.4
**$CaCl_2$**	33.7± 1.8	3.2
**$ZnCl_2$**	N.D	N.D
**$ZnSO_4$**	N.D	N.D
**$CdCl_2$**	N.D	-

Peak 1 fraction samples (no BRIL fusion) were used in measurements. Assays were performed in triplicate with standard deviations reported, where applicable. 'N.D'= activity not detected, '–' indicates no assay conducted.

Table S3 Cryo-EM Data Collection Parameters and Model Refinement Statistics

Parameters	Wildtype *Nm*qNOR (PDB ID: 6L3H, EMDB ID: EMD-0822)	Glu-494-Ala *Ax*qNOR (PDB ID: 6T6V, EMDB ID: EMD-10387)
**Microscope and Detector**	Titan Krios with K2 Summit (Gatan)	Titan Krios with K2 Summit (Gatan)
**Location**	Astbury BioStructure Laboratory (ABSL), U.K	electron Bio-Imaging Centre (eBIC), U.K
**Voltage (kV)**	300	300
**Magnification**	130,000x	48,000x
**Pixel size (Å)**	1.07	1.043
**Defocus range (µm)**	-1.5 to -3.5	-1 to -3
**Total dose (e^-/A^2)**	69.44	55.92
**No. of frames**	40	40
**Exposure time per frame**	0.3	0.3
**Dose per frame ($e^-/Å^2$)**	1.63	1.39
**No. of micrographs**	3,182	2,239
**Total autopicked particles**	972,038	684,905
**Particles in final 3D refinement**	233,556	144,424
**Symmetry Imposed**	C2	C1
**Map Sharpening *B*-factor ($Å^2$)**	-91	-278
**Map Resolution* (Å)** (FSC threshold= 0.143)	3.06	4.5
**Refinement**		
**Starting model for structure refinement**	Zn^{2+}-bound *Nm*qNOR (this work)	Val-495-Ala *Ax*qNOR (PDB ID: 6QQ6) (Gopalasingam *et al*, 2019)
**CC (mask)**	0.88	0.78
**CC (main chain)**	0.88	0.76
**CC (side chain)**	0.86	0.72
**CC (ligands)**	0.92	0.86
**Non-hydrogen atoms**	11,966	5,868
**Protein residues**	1,482	742
**Ligands**	8	3
***B*-factors† ($Å^2$)**		

**Protein**	46.89	142.38
**Ligand**	31.12	93.91
**R.M.S. Deviations**		
**Bond lengths (Å)**	0.007	0.014
**Bond angles (º)**	0.764	1.336
**Clash score**	4.30	15.31
**MolProbity score**	1.65	2.11
**Poor rotamers (%)**	0.16	0.2
**Ramachandran Plot**		
**Favoured (%)**	93.22	94
**Allowed (%)**	6.78	6
**Outliers (%)**	0	0

*Resolution determined by RELION 3.0, † Mean *B*-factors determined by Phenix

Table S4 Site directed mutagenesis of qNOR residues situated in putative proton water transfer channel

**Residue in *Nm*qNOR and % activity vs WT[a]**	**Residue in *Ax*qNOR and % activity vs WT[b]**	**Residue in *Gs*qNOR and % activity vs WT[c]**	**Residue in *Pm*qNOR and % activity vs WT[d]**
Glu-494 (-)	Glu-490-Ala (N.D)	Glu-512-Ala (N.D)	Glu-476-Asp/Gln/Ala (All N.D)
Glu-498-Ala (7 %)<br>Glu-498-Gln (20 %)<br>Glu-498-Phe (1.5 %)	Glu-494-Ala Peak 1 (5 %)<br>Glu-494-Ala Peak 2 (N.D)	Glu-516-Ala (-)	Glu-480-Asp (195 %)<br>Glu-480-Gln (N.D)
Asn-604 (-)	Asn-600-Ala (7 %)	Asn-622 (-)	Asn-586-Leu (N.D)
Ala-527-Phe (N.E)	Ser-523-Ala (30 %)	Gln-545 (-)	Glu-509-Leu (58 %)
Glu-573-Phe/Glu-259-Gln (70%)<br>Glu-573-Phe/Glu-259-Leu (80 %)	Glu-569-Ala (N.E)	Glu-591 (-)	Glu-555-Leu (80 %)

(-) = No mutagenesis attempted

(N.D) = Activity not detected

(N.E.) = No over-expression of variant

a= data from Gonska *et al.,* 2018

b= data from Gopalasingam *et al.,* 2019

c= data from Matsumoto *et al.,* 2012

d= data from Sheraden, 2013

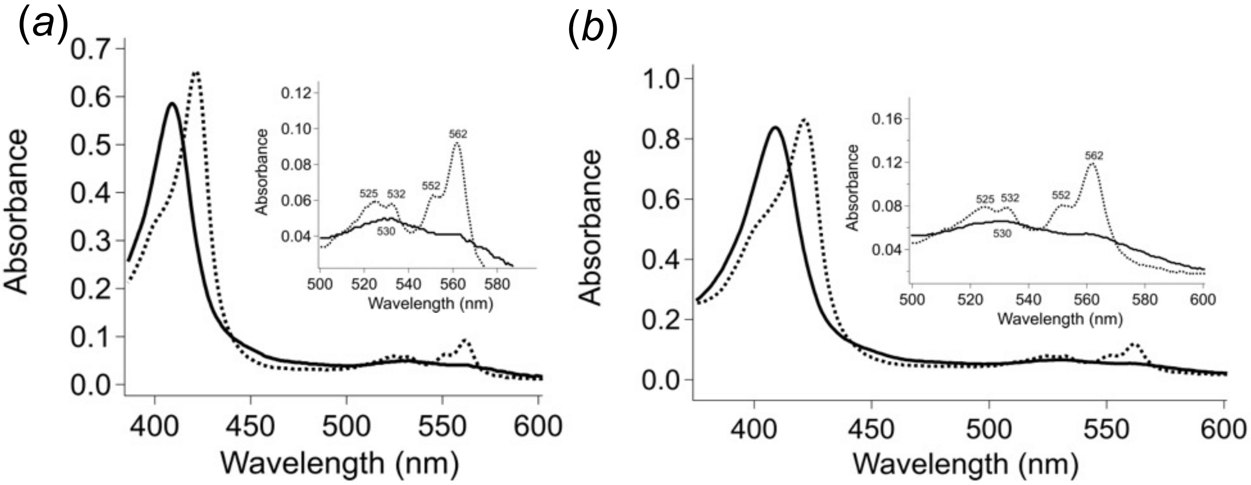

Figure S1 UV visible absorption spectra of *Nm*qNOR chromatographic fractions. Oxidized (solid curve) and dithionite reduced forms (broken curve) spectra are shown for peak 1 (*a*) and peak 2 (*b*) chromatographic fractions following purification, with the Q-band region of the spectra shown inset. Proteins were stored in 50 mM HEPES pH 8.0, 150 mM NaCl, 0.05 % (v/v) DTM. Spectra are consistent with previous data from Gonska., *et al*, 2018.

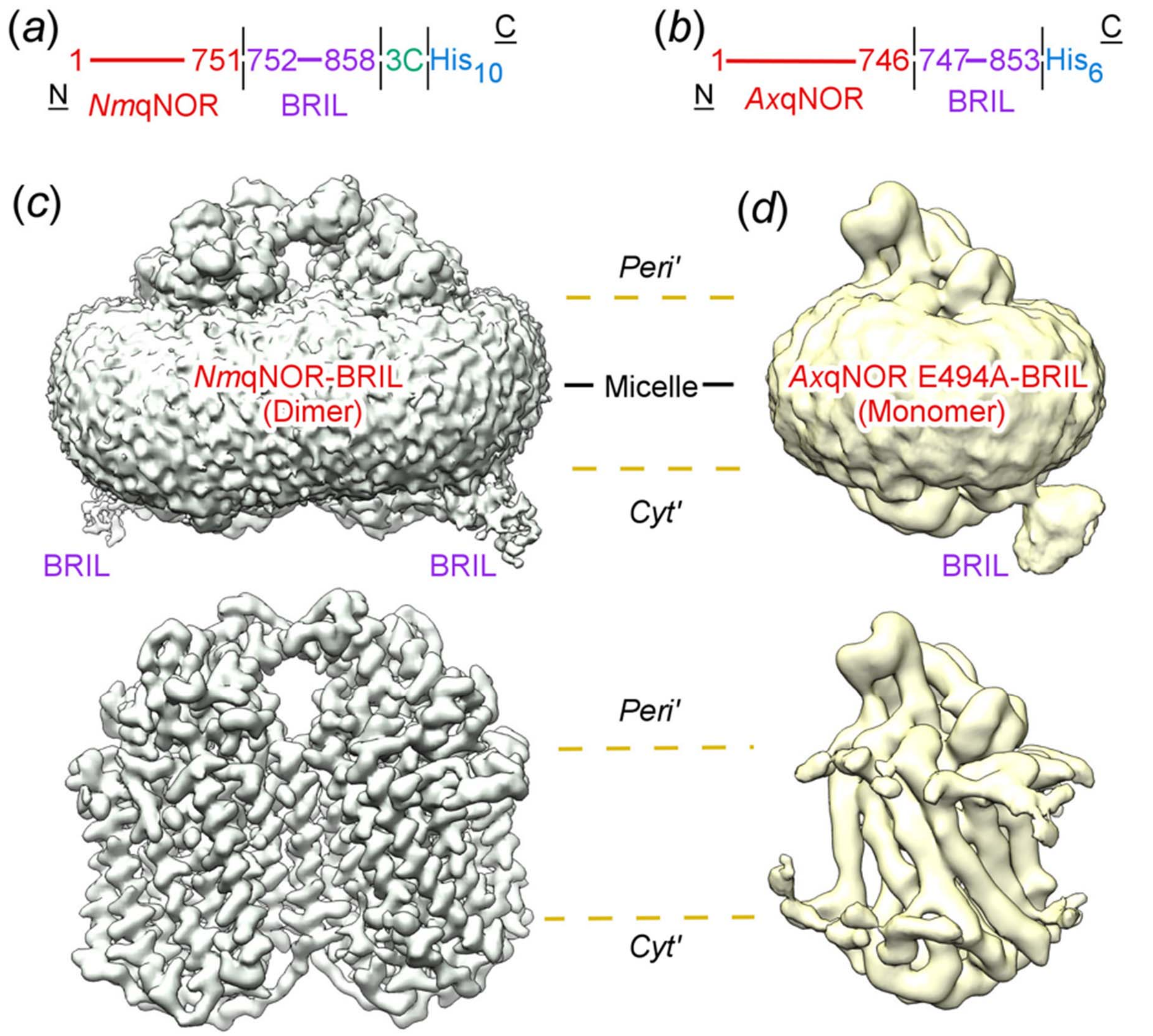

Figure S2 qNOR-BRIL unsharpened density maps. (*a*-*b*) Construct layout for *Nm*qNOR-BRIL and E494A *Ax*qNOR-BRIL. *Nm*qNOR-BRIL contains a 3C protease cleavage site ('3C'), to facilitate cleavage of the decahistidine tag. N- and C-termini are indicated with N and C, respectively. (*c*) *Nm*qNOR-BRIL cryo-EM dimer reconstruction shown at two thresholds, the top map (0.0063 binarisation threshold) shows the BRIL portion of the reconstruction on the cytoplasmic (*Cyt'*) side. The bottom map is shown at 0.0125 binarisation threshold, without the micelle and BRIL molecules. (*d*) E494A *Ax*qNOR-BRIL cryo-EM monomer reconstruction shown at two thresholds, the top map (0.0078 threshold) shows the BRIL portion, with the bottom map (0.0118 threshold) shown without the micelle and BRIL. Dashed yellow line demark cytoplasmic and periplasmic (*Peri'*) sides. Map threshold values taken from UCSF Chimera v.1.14, with the 'Hide dust' tool employed in figures (*c*) & (*d*) to remove residual spots of micelle density for clarity.

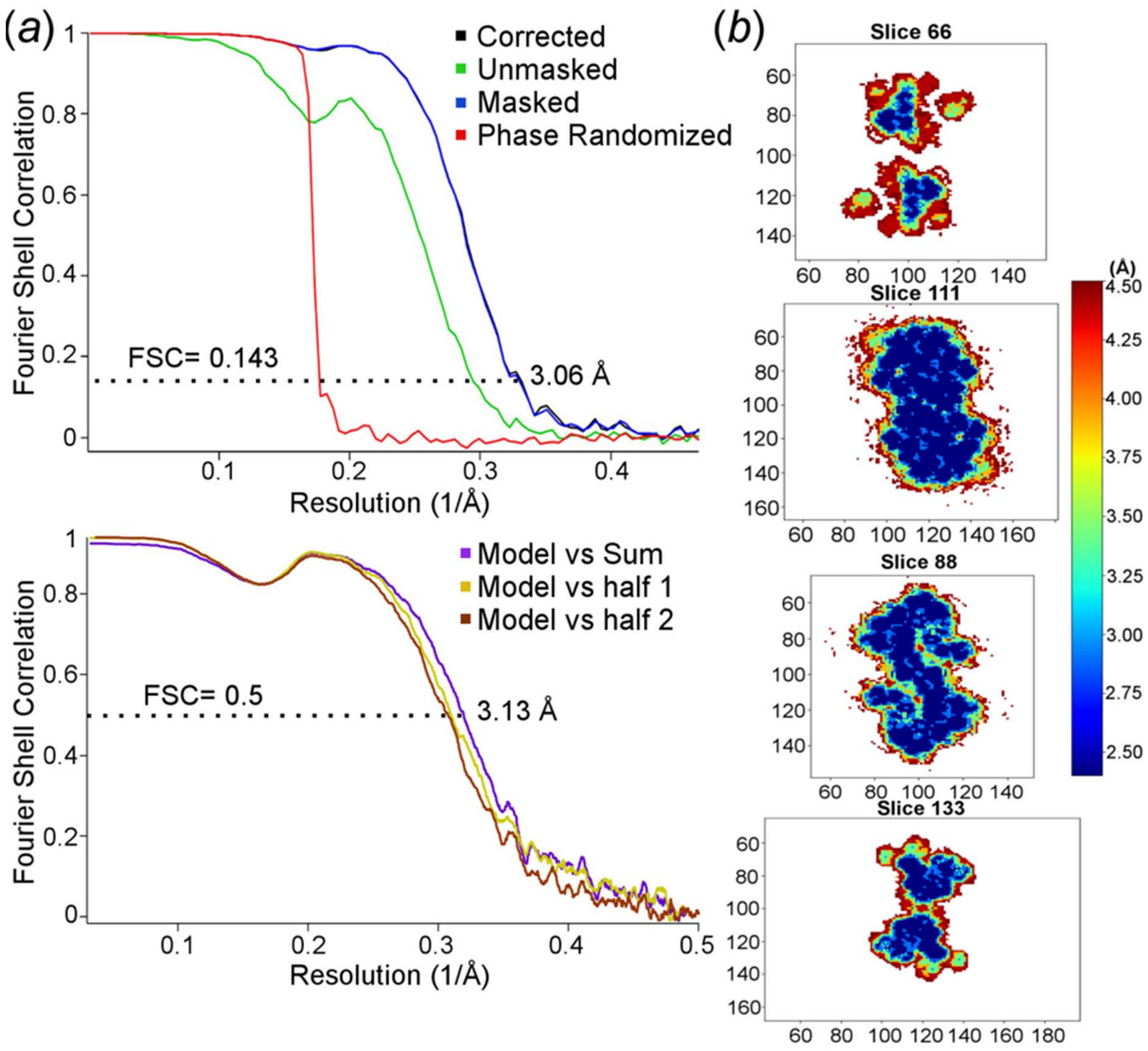

Figure S3 Cryo-EM refinement and validation of *Nm*qNOR-BRIL. (*a*) Top, FSC curves (correlation corrected= black, unmasked maps= green, masked maps= blue and phase randomized masked maps= red) of *Nm*qNOR-BRIL cryo-EM reconstruction, with 'gold standard' FSC= 0.143 shown as a broken, black line. Bottom, map-model correlations of summed map/FSC_{sum} (purple), half 1 map/FSC_{work} (gold) and half 2 map/FSC_{free} (brown), with FSC= 0.5 shown as a broken, black line. Validation performed by Mtriage, within Phenix. (*b*) Slice through local resolution estimation performed by ResMap, using the default settings, with orthogonal slices of the map coloured by a resolution gradient scale (right).

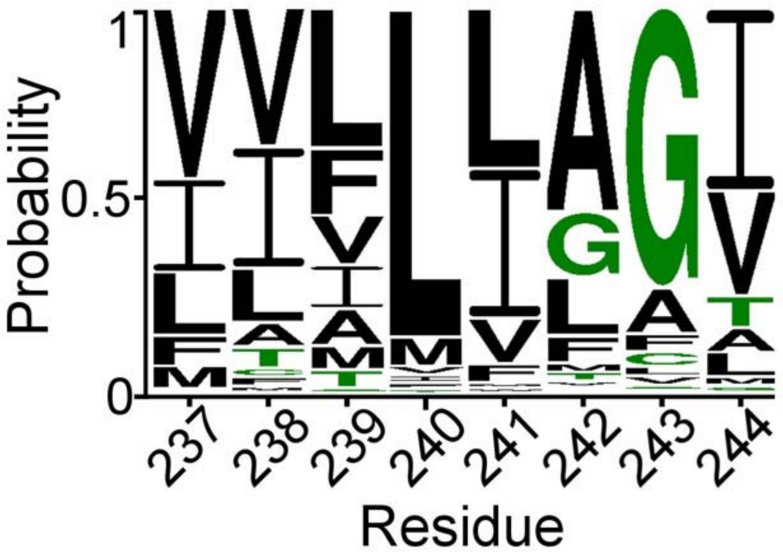

Figure S4 Consensus sequence of TMII residues in qNORs**.** Four amino acids residues (Val-237, Leu-240, Leu-241 and Ile-244) that are involved in dimerization of *Nm*qNOR are well conserved (> 40 % conservation) amongst the 224 qNOR sequences analyzed. Figure prepared using WebLogo v3.0 (Crooks *et al*, 2004). Probability refers to probability of residue occurrence at the specified sequence position (x-axis).

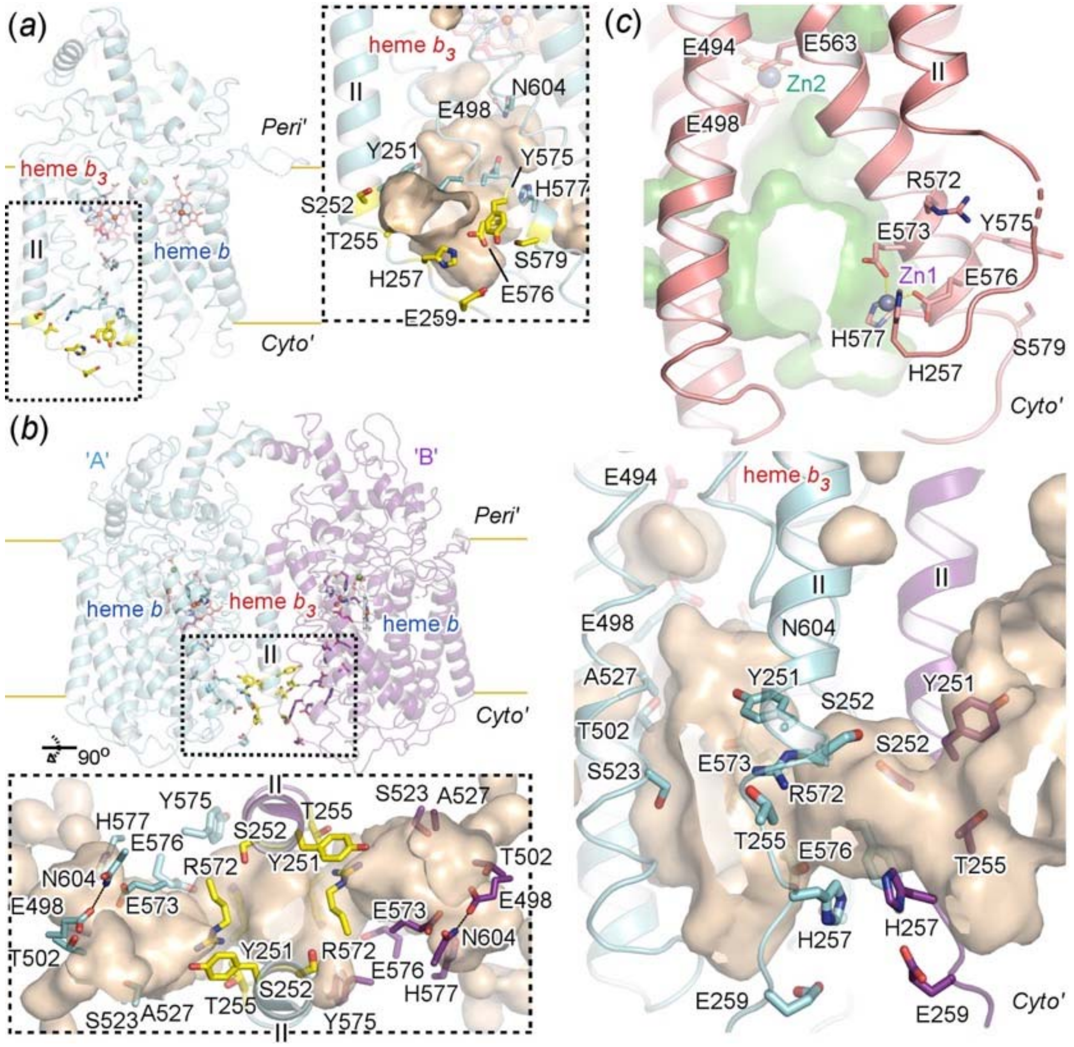

Figure S5 Putative proton transfer water channel cavities within *Nm*qNOR cryo-EM derived dimeric structure. (*a*) Monomer portion from the dimer in plane of membrane (left), with yellow lines marking the periplasmic (*Peri'*) and cytoplasmic (*Cyto'*) ends. Residues Ser-252, Thr-255, Glu-259, Tyr-575, Glu-576 and Ser-579 (yellow sticks) may form the start of the putative proton water transfer channel (black dashed box) from the cytoplasmic end, right, zoomed in portion of the black dashed box, with the cavity (brown surface) originating from aforementioned residues, which travels towards the binuclear center (heme b_3 and Fe_B). (*b*) Left, dimer in plane of membrane, coloured by chain; 'A' in cyan and 'B' in purple cartoon, respectively. TMII marked as roman numeral and residues that may partake in synergising proton transfer pathways between each chain are shown as yellow sticks (within black dashed box). Bottom, cavity (brown surface) viewed from the periplasmic side, showing connectivity between the two chains. Several residues (Tyr-251, Ser-252, Thr-255, and Arg-572) may help maintain connectivity between both monomers and help form the putative proton transfer water channel (shown by purple and cyan sticks, respectively) toward the Glu-498 and Asn-604 'junction' and active site (further details in [Fig. 4(*d*)]. *(c)* Top, *Nm*qNOR Zn-bound crystal structure putative

proton transfer channel (cavity shown as green surface), showing how Zn1 binding site may cause cavity disruption on the cytoplasmic side and perturbing TMII dimer stabilisation, compared to the dimeric, active *Nm*qNOR cryo-EM structure in the same orientation (bottom). TMII provides a cavity (brown surface) connection between respective chains. PyMOL cavity detection radius= 3 solvent radii and cavity detection cutoff= 4 solvent radii.

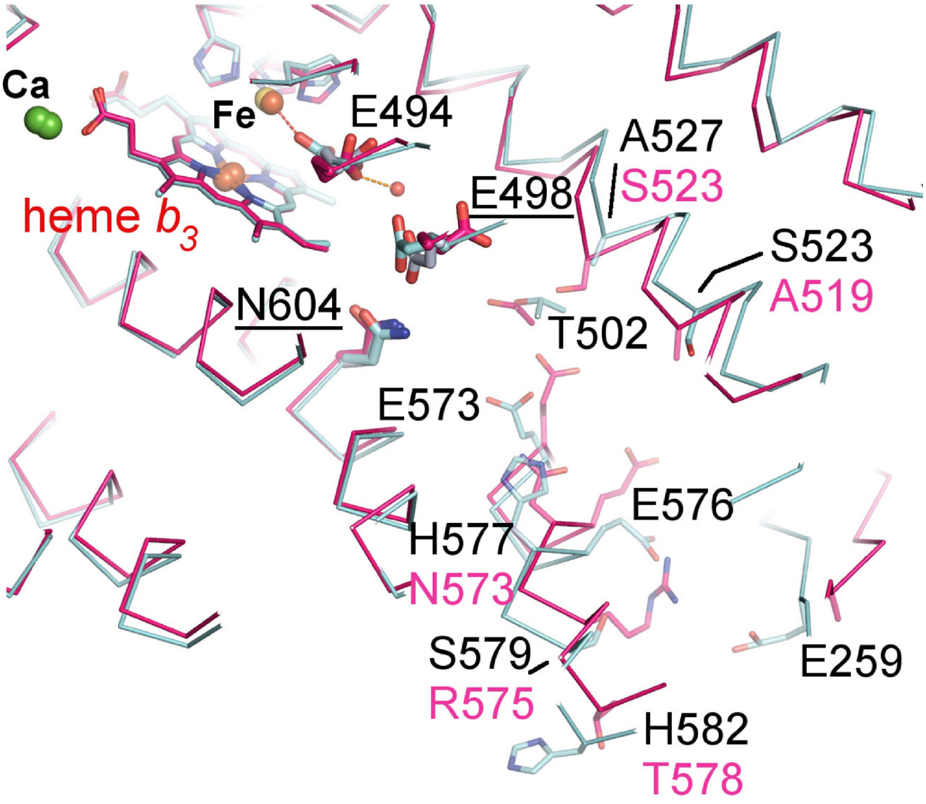

Figure S6 Putative proton transfer channels amongst *Nm*qNOR and *Ax*qNOR cryo-EM structures. *Nm*qNOR (cyan ribbon and sticks, residues labelled in black) and *Ax*qNOR (magenta ribbon and sticks for Val-495-Ala structure, grey sticks for wildtype, differing residues labelled in magenta) display more conformational and structural heterogeneity in the first half of the putative proton transfer channel, e.g. from E259 till T502 (*Nm*qNOR numbering). N604 and E498 show conserved conformations (aside from in V495A for the latter, due to increased flexibility) and may form a 'junction' for protons to pass through on toward E494 and the active site. Note, in *Ax*qNOR structures of wildtype and Val-495-Ala (both active), terminal glutamate does not ligate Fe_B, yet in *Nm*qNOR this glutamate does ligate Fe_B (brown sphere).

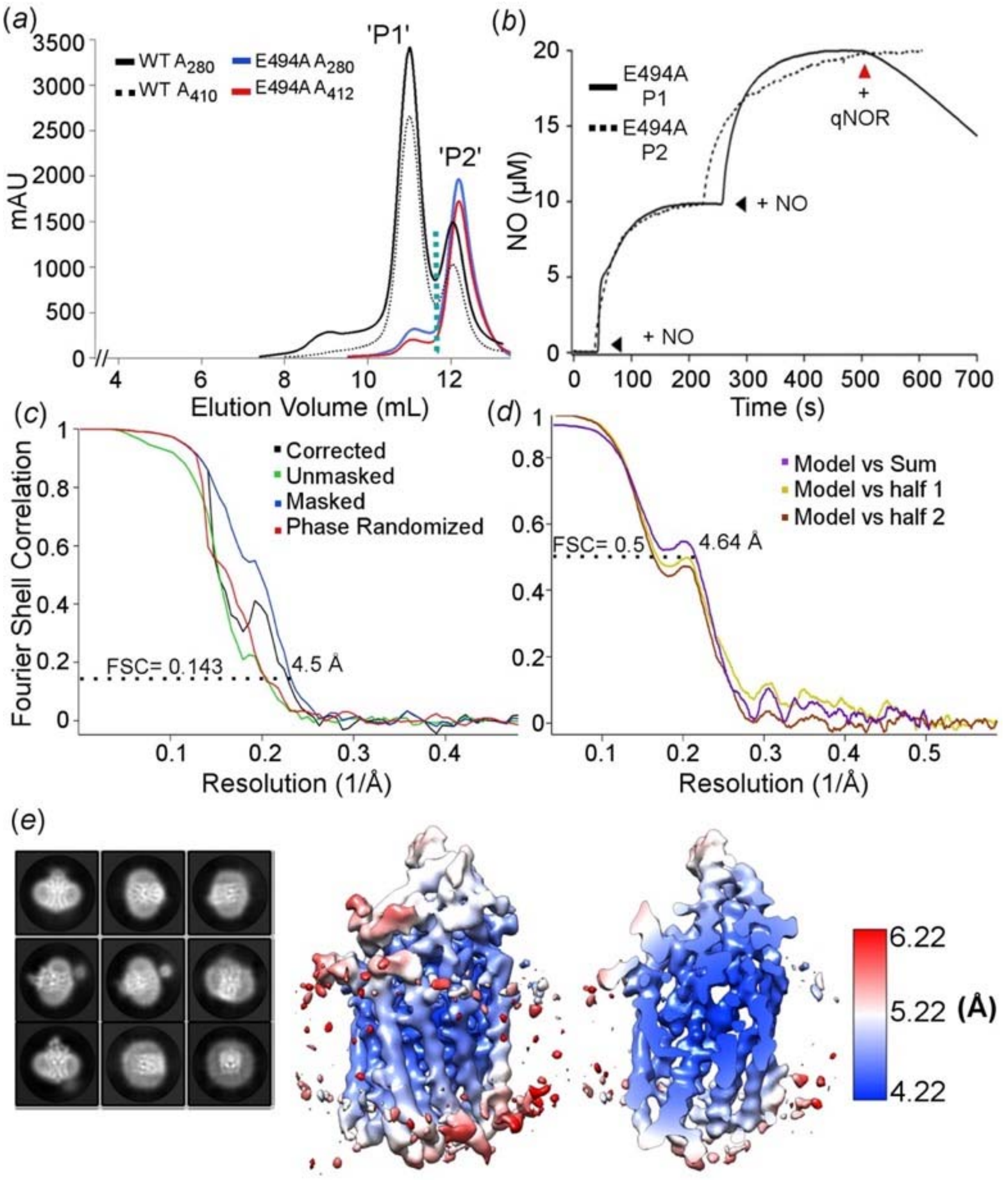

Figure S7 *Ax*qNOR Glu-494-Ala-BRIL characterization and cryo-EM analysis. (*a*) Gel filtration profile of *Ax*qNOR wildtype-BRIL (solid and dashed black lines) compared to E494A-BRIL (solid blue and red lines). Green dashed line indicates samples of peak 1 (P1) and peak 2 (P2). Purified samples were run down a Superdex 200 10/300 Increase (GE Healthcare) in 50 mM Tris pH 7.5, 150 mM NaCl, 0.05 % (v/v) DTM, (*b*) NO consumption curves for E494A, with peak 1 (solid line) exhibiting 5 % activity compared to wildtype, with peak 2 (dashed line) showing no consumption of NO. Black triangles indicate NO addition and red triangle indicates qNOR addition (final concentration of 0.05 μM), (*c*) FSC curves (correlation corrected= black, unmasked maps= green, masked maps= blue and phase randomized masked maps= red) of E494A-*Ax*qNOR-BRIL monomer cryo-EM reconstruction, with 'gold standard' FSC= 0.143 shown as a broken, black line. (*d*) Map-model correlations of summed map/FSC_{sum} (purple), half 1 map/FSC_{work} (gold) and half 2 map/FSC_{free} (brown), with FSC= 0.5 shown as a broken, black line. Validation performed by Mtriage, within Phenix. (*e*) Left, 2D class averages of polished particles (box size of 150 pixels) , middle and right;

3D EM density map of E494A-BRIL P2 filtered and coloured by resolution (density for detergent micelle and BRIL hidden), according to colour key on the right hand side. Resolution estimated within RELION 3.0.

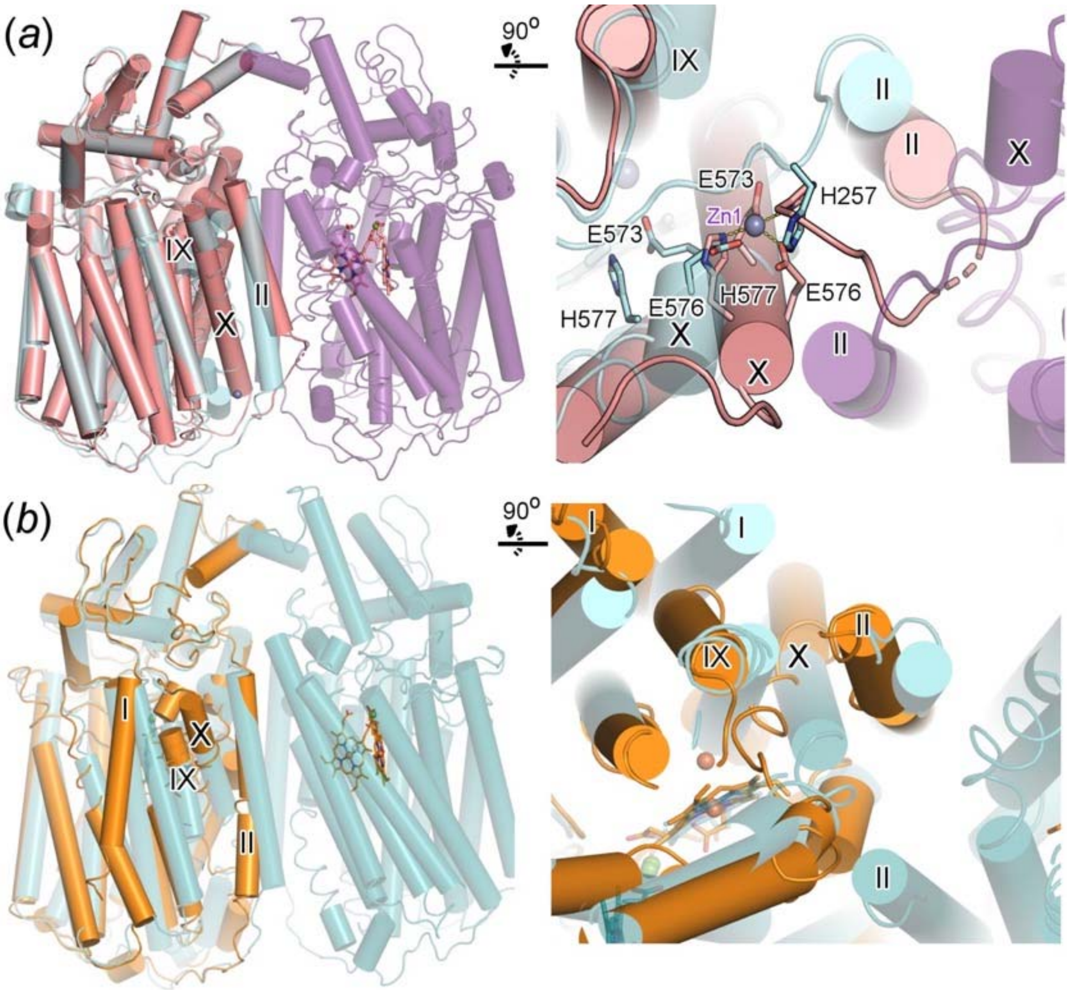

Figure S8 Structural comparisons between monomer and dimer structures of qNORs. (*a*) Left, alignment of *Nm*qNOR monomeric, Zn bound structure (salmon cylinders) against the cryo-EM dimeric structure of *Nm*qNOR (cyan and purple cylinders). TMII, IX and X marked as roman numerals. Right, view from cytoplasmic end shows the distortion of monomeric TMII (salmon) away from TMXI, with the loop clashing into TMX of the symmetry related monomer (purple cartoon) of the cryo-EM derived dimer. Zn1 (grey sphere) binding may cause this distortion and contribute to dimer-monomer transition, with Zn1 binding ligands shown as salmon sticks. (*b*) Left, alignment of *Ax*qNOR cryo-EM dimer (teal cylinders) against the Glu-494-Ala monomeric cryo-EM structure (orange cylinders), with TMI, II, IX and X marked. Right, cytoplasmic end view of helical rearrangements, with the largest shifts occurring in ‘broken’ TMIX and X, which flank the proposed proton transfer channel.

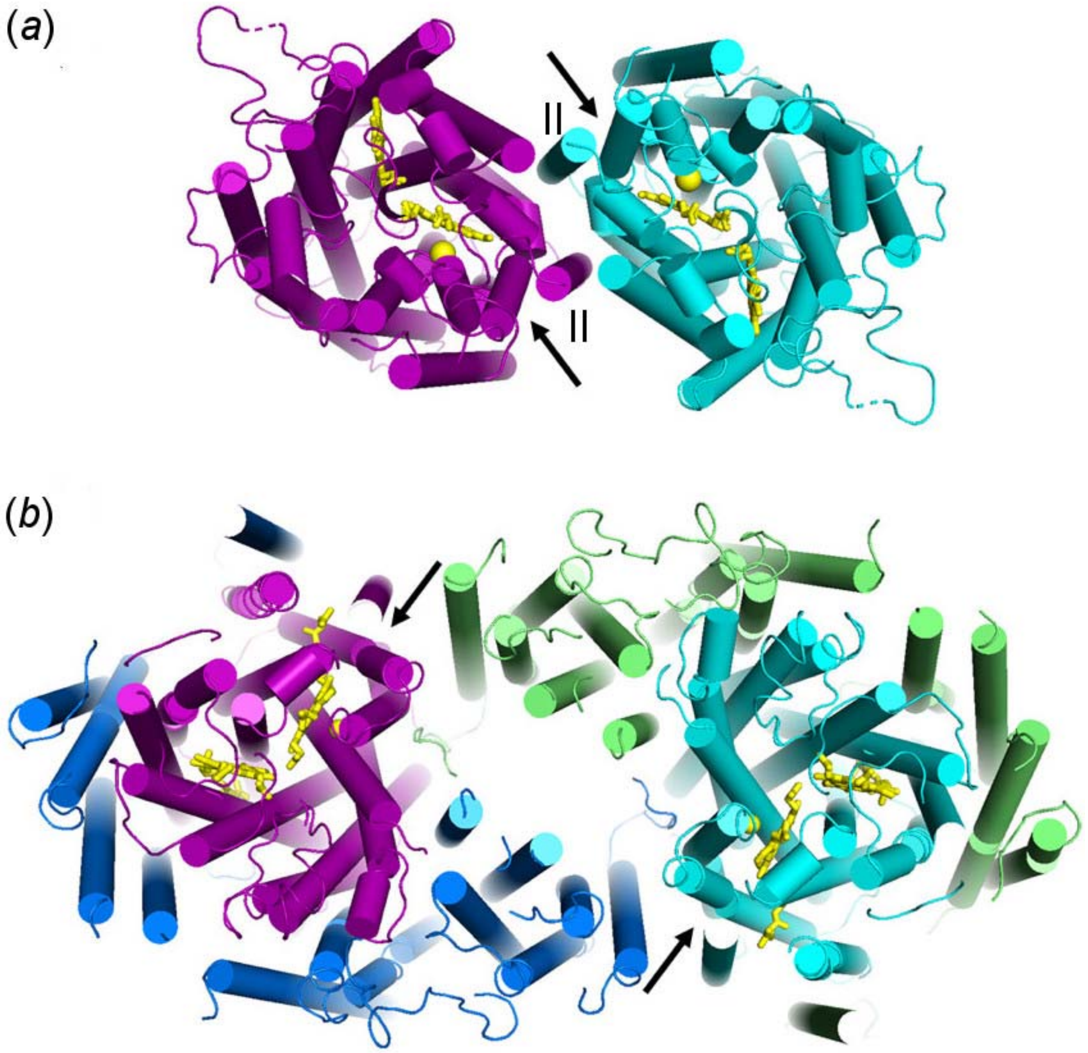

Figure S9 Comparison of dimeric structures between *Nm*qNOR and bovine C*c*O. (*a*) *Nm*qNOR dimer coloured by chain, A in purple and B in cyan cartoon, respectively. Hemes *b* and b_3 and Fe_B are colored in yellow. Entry point of the proton transfer water channels are shown by black arrows and TMII marked (roman numerals). (*b*) Bovine C*c*O dimer (PDB ID: 1V54) coloured by chains, A and B in purple, and N and O in cyan cartoon, respectively. Other chains are colored in blue and green. Hemes *a* and a_3 and Cu_B are colored in yellow. Entry point of the proton transfer K-pathways are shown by black arrows.
